# Landscape evolution under the southern Laurentide Ice Sheet

**DOI:** 10.1126/sciadv.abj2938

**Published:** 2021-11-24

**Authors:** Shawn Naylor, Andrew D. Wickert, Douglas A. Edmonds, Brian J. Yanites

**Affiliations:** 1 Center for Geospatial Data Analysis and Indiana Geological and Water Survey, Indiana University, Bloomington, IN 47405, USA.; 2 Department of Earth and Environmental Sciences and Saint Anthony Falls Laboratory, University of Minnesota, Minneapolis, MN 55455, USA.; 3 Department of Earth and Atmospheric Sciences, Indiana University, Bloomington, IN 47405, USA.

## Abstract

Subglacial landscapes, revealed in regions of recent ice-sheet retreat, provide a window into ice-sheet dynamics and interactions with evolving subglacial topography. Here, we document landscape evolution beneath the southern Laurentide Ice Sheet of North America since the end of the Pliocene, 2.6 million years (Ma) ago, by reconstructing the isostatically adjusted preglacial surface and modern bedrock topography at 250 m horizontal resolution. We use flow routing to reconstruct drainage networks and river longitudinal profiles, revealing the pattern and extent of their glacially forced reorganization. The overall mean Quaternary (2.6 Ma ago to present) erosion rate is 27 m/Ma, rising within ice-streaming corridors to 35 m/Ma (and locally reaching 400 m/Ma) and falling to 22 m/Ma in non–ice-streaming regions. Our results suggest that subglacial erosion was sufficient to lower the southern Laurentide Ice Sheet into warmer environments, thereby enhancing ablation and reducing ice-sheet extent over time.

## Teaser

Ancient glaciated landscapes reveal interactions among ice dynamics, bed composition, and preglacial river networks.

## INTRODUCTION

During the Quaternary, the Laurentide Ice Sheet (LIS) repeatedly advanced across the North American mid-continent, modifying the landscape and depositing low-relief and agriculturally productive sediments across the north-central United States and southern Canada. Buried beneath these deposits lies a hidden topography of river gorges, drainage divides, and glacially scoured bedrock that records the past 2.6 million years (Ma) of landscape evolution (*1*–*6*). Understanding this landscape and its development is critical for accurately reconstructing the dynamics and history of the LIS, which researchers often use to examine the long-term behavior of ice-sheet models, and to understand the feedbacks between landscape and ice dynamics (*7*–*9*). Such feedbacks are hypothesized to have caused the mid-Pleistocene transition (*10*), generate marine ice-sheet instability (*11*, *12*), and result in extensive Early Pleistocene glacial advances (*13*).

Existing glacial deposits and modeling provide direct evidence for at least 10 LIS advances and retreats in the Quaternary (*14*) that transformed the landscape and river systems across North America in the process. While the effects of some of these LIS advances and retreats can be isolated, their cumulative impact on landscape change is largely unknown because the initial end-Pliocene topography, which strongly determined the trajectory and pace of landscape evolution (*9*, *10*), has never been reconstructed.

The rearrangement of river networks during the Quaternary was accomplished through erosion and deposition of material during LIS advances and retreats, but understanding the interplay between the LIS and North America’s landscape evolution is difficult without maps of bedrock erosion. Glacially scoured landscapes of the Laurentide region are intuitively associated with erosion, but most estimates of glacial denudation are inferred from volumes of offshore Quaternary sediments (*2*), observations of surficial geology (*3*, *15*, *16*), or modeling studies, whose authors note the sparse and/or inconsistent data available for validation (*17*, *18*). These different inferences led to total erosion estimates throughout the Quaternary ranging from 1 m to tens of meters (*3*, *15*, *18*) to hundreds to thousands of meters (*2*, *4*, *17*). In this study, we directly assess the integrated effects of LIS advances and retreats on erosion and deposition by using our modern, bedrock, and Pliocene surface digital elevation models (DEMs).

## RESULTS

### Mapping Quaternary bedrock erosion

**Fig. 1.**
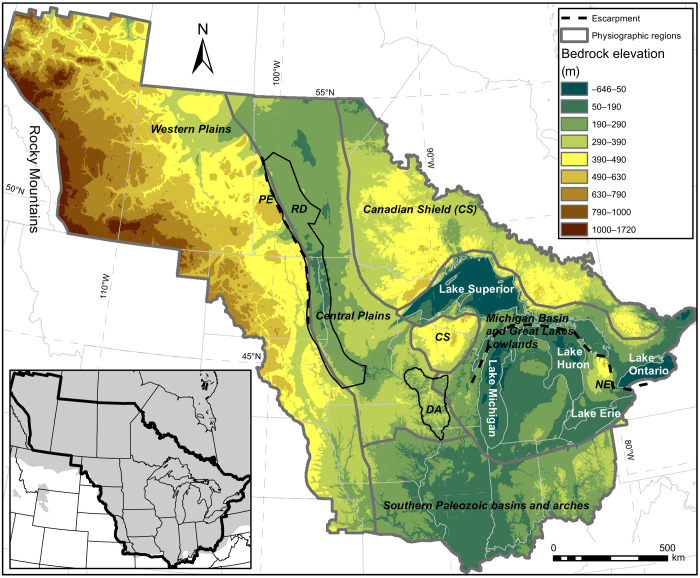
Bedrock topography of the Laurentide region. Gray outlines mark physiographic provinces, and black lines denote the Driftless Area (DA), Red River Valley/Des Moines Lobe region (RD), and the Pembina (PE) and Niagara (NE) Escarpments. The inset map shows the analysis extent (black outline) atop the mapped maximum Quaternary ice extent (gray shading) (*14*).

**Fig. 2.**
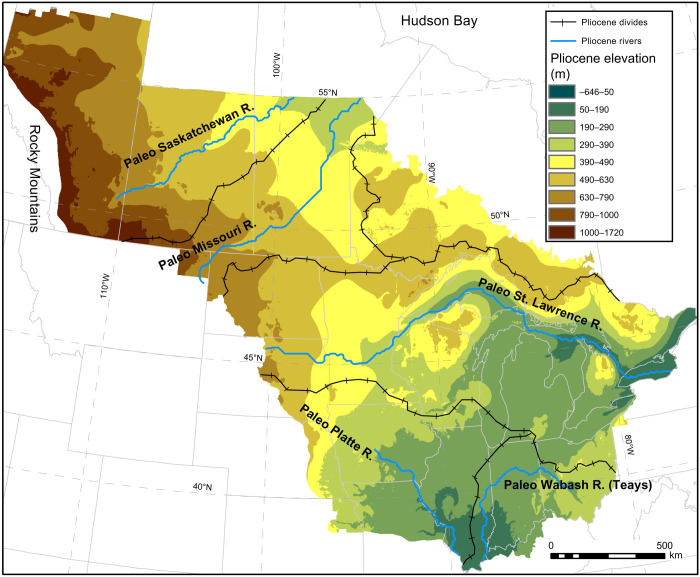
Pliocene surface reconstruction with paleo-drainage. We extracted paleo-watershed boundaries and river longitudinal (long) profiles following the five dominant paleo-rivers denoted in solid blue lines.

We map landscape change through the southern LIS region by reconstructing two key surfaces. First, we create a seamless 250-m-resolution grid of the bedrock surface of the North American mid-continent (Fig. 1) using depth-to-bedrock data derived from residential and municipal water well logs, drill cores, geotechnical borings, and seismic surveys. Second, we reconstruct preglacial (i.e., end-Pliocene) paleotopography (Fig. 2) by linking our bedrock topography (Fig. 1) with (i) paleo-drainage evidence contained in that surface and from the published literature and (ii) known regions of little to no erosion or deposition (see Materials and Methods). Our end-Pliocene DEM is an interpolated surface from newly reconstructed preglacial drainage patterns, valley-bounding strath terraces from 187 valley cross sections, relict landforms, and computed residual glacial isostatic adjustment (GIA) (see Materials and Methods and figs. S3 to S6) (*19*). From this DEM, we use flow-routing algorithms to quantitatively reconstruct paleo-drainage basins and river courses (Fig. 2). Together, these portray a landscape and hydrography with integrated drainage and more extensive north- and east-flowing river systems, in contrast to today’s glacially altered landscape.

By subtracting the bedrock surface from the Pliocene surface, we find that over the course of the Quaternary, the southern LIS region experienced 71 m of spatially averaged bedrock erosion (27 m/Ma). This is almost double the average continental erosion rate (16 m/Ma) during the Phanerozoic (*20*). Similarly, subtracting the bedrock surface from the modern surface indicates an average of 39 m of deposition. Although glacial landforms dominate much of the region, these spatially averaged estimates also include erosion associated with glacial, paraglacial, and proglacial processes.

### Spatial patterns of glacial erosion

**Fig. 3.**
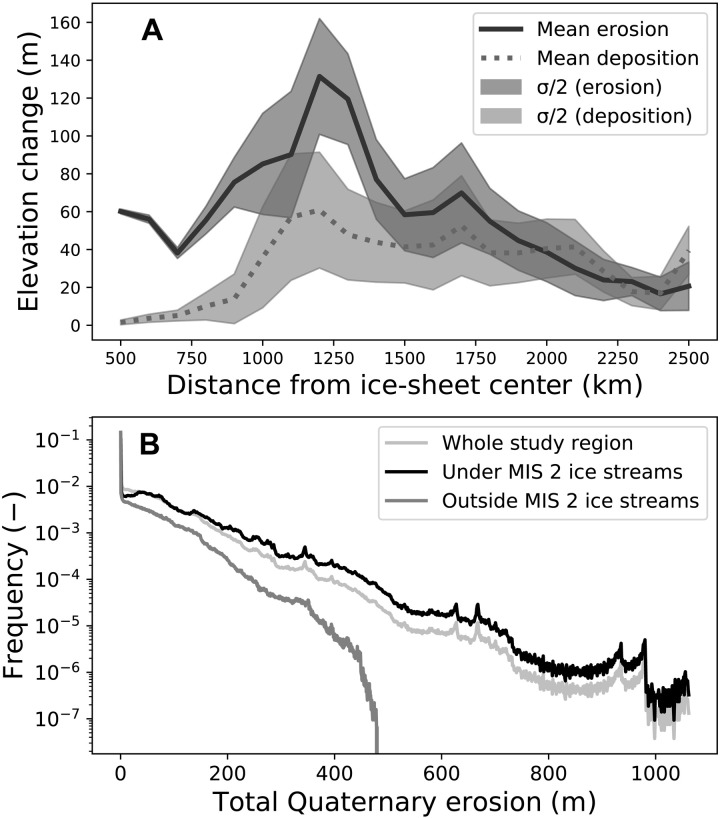
Erosion and deposition within the study area. (**A**) Erosion and deposition relative to the LIS domes and divide in and around Hudson Bay (*21*). Erosion is calculated by subtracting the end-Pliocene surface from the bedrock surface. Deposition is calculated by subtracting the modern surface from the bedrock surface. Mean values of erosion and deposition are binned every 100 km. See figs. S7 and S8 for distance from ice-sheet center contours overlain on the study area, and for reference, the middle of Lake Superior is located approximately 1200 km from the ice-sheet saddle. (**B**) Bedrock erosion histograms normalized to total erosion in the study region. Deep erosion occurred only under mapped zones of streaming ice (*63*), which we infer to be persistent ice-streaming regions throughout much of the Quaternary.

**Fig. 4.**
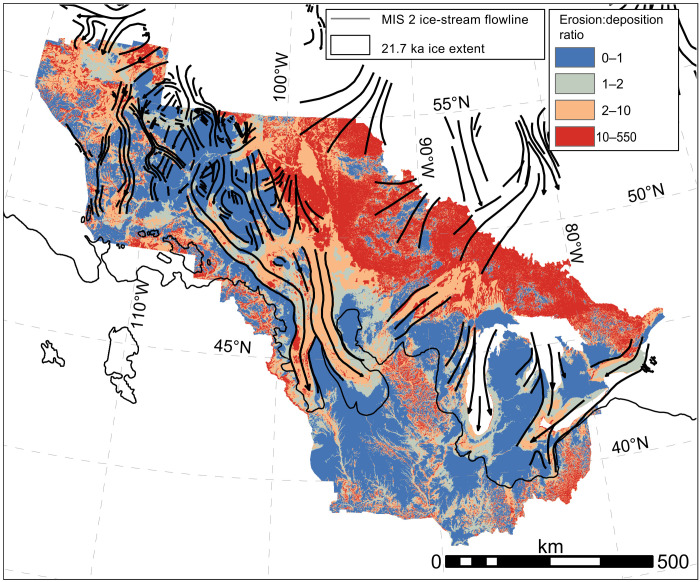
Erosion-to-deposition ratio. Previously mapped major marine isotope stage 2 (MIS 2) ice-stream flowlines (*63*) and ice extents (*64*) are plotted atop a color map indicating the ratio of bedrock erosion (end-Pliocene elevation minus modern bedrock elevation) to sediment thickness (modern ground-surface elevation minus modern bedrock elevation).

Erosional and depositional maxima in our study area occur 1200 km from the Hudson Bay ice saddle, which connected the Keewatin and Québec-Labrador domes of the Last Glacial Maximum LIS (Fig. 3A and figs. S7 and S8) (*21*). The position of these zones coincides with empirical evidence (*22*) and the modeled presence (*17*, *23*) of fast-moving, warm-based ice during the last 120,000 years. Farther down-ice from this maximum, erosion and deposition become indistinguishable except at the southern edge of the study area (Fig. 4), in a pattern consistent with idealized physics-based modeling (*24*). However, in contrast to the modeling, our data demonstrate that erosional and depositional maxima both lie up-ice from the southernmost ice-sheet margin. This may be reconciled by acknowledging that the LIS often was smaller than its maximum extent (*14*) and that the observed erosional maximum lies at the transition from Canadian Shield bedrock to more erodible sedimentary units (*25*).

Alpine glacial erosion rates are proportional to the square of ice-sliding velocity (*26*), and a comparison of streaming versus nonstreaming zones of the LIS supports velocity-dependent erosion rate laws for continental ice sheets as well. Outside of ice-streaming corridors, the southern Laurentide region experienced 57 m (22 m/Ma) of spatially averaged erosion during the Quaternary. In contrast, ice-streaming corridors experienced 91 m of erosion (35 m/Ma), comparable with previous estimates of spatially distributed LIS bedrock erosion (120 m) based on off-shore sedimentation (*2*). Ice streaming produced deep scours of up to ~1 km (Fig. 1 and fig. S8). The ~400 m/Ma erosion rates in the deep scours of the LIS are typical for valley glaciers (*27*), plausible for tectonically active mountain belts (*28*), and extreme compared to long-term exhumation rates for the North American Craton (*29*). At the margin of the ice sheet, our glacial erosion-rate estimates are conservative because ice did not continuously occupy this area and some of the erosion may be caused by subaerial fluvial processes. In the Great Lakes basins, where we measured maximum erosion rates, our estimates are more accurate.

Volumetrically, 60% of the total erosion occurs in ice-streaming regions, which cover 40% of the study area (1.34 million km^2^), and the highest rates of erosion are dominantly or only seen here (Fig. 3B). Beneath ice streams, erosion depths are exponentially distributed (Fig. 3B), similar to findings from subglacial bedforms (*30*), and suggesting that this could be a fingerprint of glacial erosional processes. Outside of ice-streaming regions, this exponential erosion rate distribution is truncated. The 14:1 ratio of deep scour to mean erosion matches interpretations of the subglacial topography of Antarctica (*7*).

Quaternary deposition averaged 39 m (15 m/Ma), bracketed by mean deposit thicknesses of 38 m in ice-streaming regions and 40 m outside of them. Deep scours, where erosion:deposition ratios can exceed 10:1 (Fig. 4), may form with the assistance of sediment that protects their margins from quarrying and abrasion (*31*). Likewise, sediment deposits outside of ice-streaming regions, whose erosion:deposition ratio is closer to 3:2, may protect the underlying bedrock from erosion (*31*), further enhancing the erosion–ice-streaming feedback and associated nonuniform rates of erosion.

### Preglacial river long profiles and Quaternary drainage alterations

**Fig. 5.**
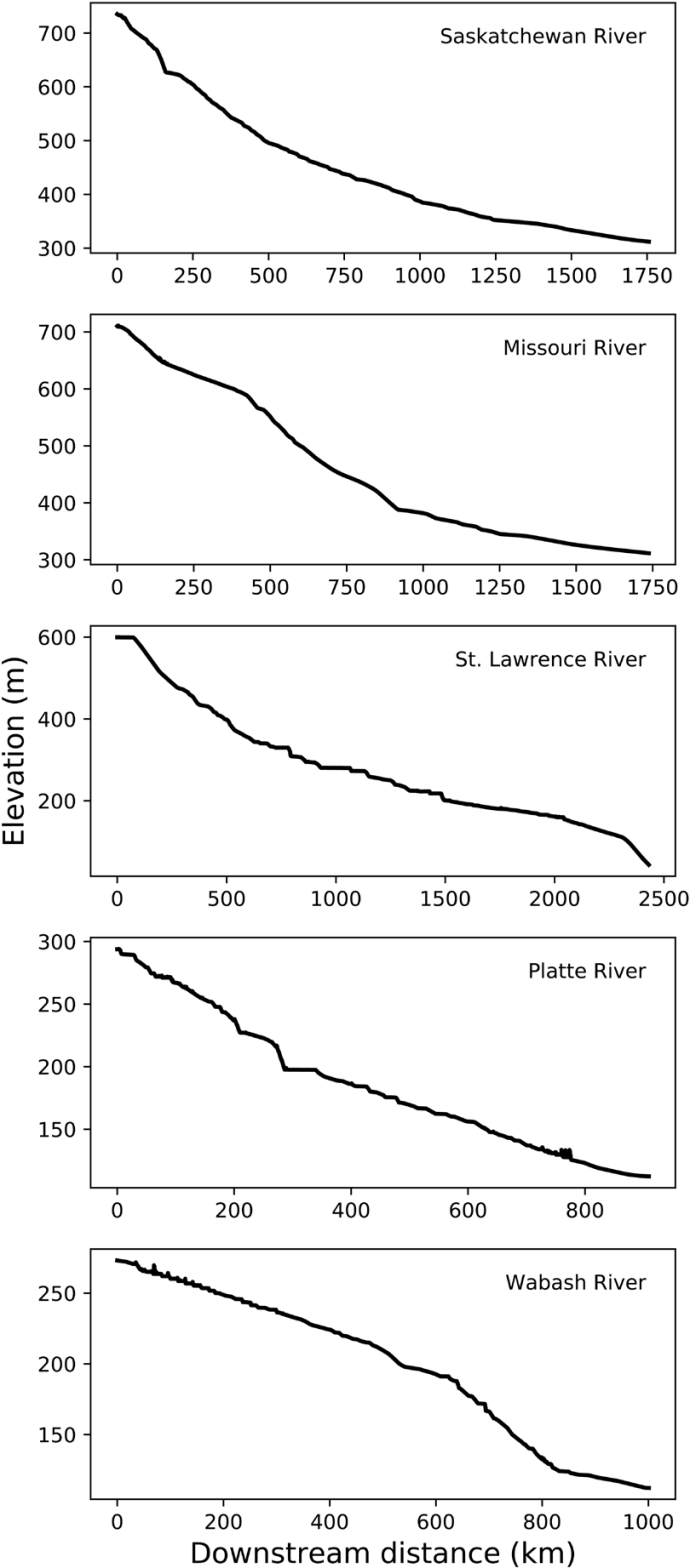
Longitudinal (long) profiles for major preglacial river networks extracted from the Pliocene DEM. Most river long profiles are concave. The smallest river in our analysis, the Wabash, bears a convexity that could relate to complexities associated with the variable rock type in the region (*65*) or mid-continental seismicity that has been shown to correlate with river morphology (*66*). See Fig. 2 for locations of main paleo-river channels along which profiles were extracted.

Most reconstructed rivers in our end-Pliocene DEM bear concave-upward longitudinal (long) profiles, typical of graded rivers (Fig. 5) (*32*). Furthermore, our geomorphically reconstructed river courses align with the sedimentologically reconstructed northern boundary of the pre-Quaternary Mississippi River drainage basin (*5*). These end-Pliocene river long profiles provide a dataset that is more appropriate than the modern glacially altered land surface and rivers for past reconstructions of uplift patterns (*33*) and dynamic topography (*34*).

Ice advance rerouted northward- and eastward-flowing rivers by damming them, forming lakes that overtopped and incised drainage divides (fig. S9) with the aid of abundant glacial meltwater (*6*). On a continental scale, this process integrated the modern Mississippi River system (*1*, *6*, *35*), redistributing water and sediment discharge across the landscape and to the sea (*1*). More locally, buried and exposed bedrock valley systems record multiple phases of glacially mediated river rerouting. In the Western Plains, narrow, steep-walled, north-south–oriented valleys cross both the preglacial drainage system (Figs. 2 and fig. S5) and one another. These valleys lack well-developed tributary networks, indicating that glaciers (or their impacts on drainage systems) (*36*), and not precipitation, provided the dominant source of water that carved them. In the Central Plains, the south-flowing Mississippi crosscuts preexisting northeast-oriented drainages and their associated bedrock cuestas (*1*).

## DISCUSSION

Both the modern surface and bedrock topography of the southern LIS region bear the characteristics of a positive feedback between glacial erosion and ice streaming (*37*) mediated by regional variations in bedrock erodibility. The Canadian Shield basement, comprising Precambrian igneous and high-grade metamorphic rocks, was exhumed by glacial erosion of sedimentary cover. As ice crossed onto thicker sedimentary rock packages—including shallow marine sediments of the Western and Central Plains, syn-rifting sediments in and around the Lake Superior basin, and Paleozoic bedrock through the Michigan basin and Great Lakes Lowlands—it preferentially eroded through weaker lithologic units (*38*). Increased ice thickness and velocity in these eroded regions accelerated their erosion in a feedback loop, forming the overdeepened basins of the Great Lakes (*39*) and the large ice-streaming corridors on the Western and Central Plains (*40*). This erosion–ice-flow feedback preserved the Niagara escarpment, which bounds the Great Lakes ringing the Michigan basin and served as a divide between ice lobes, and most likely prevented the glaciers from entering the Driftless Area (*41*) of Wisconsin and northwestern Illinois (Fig. 1).

Quaternary erosion in the southern Laurentide region likely affected ice-sheet dynamics by enhancing ablation. Early Pleistocene LIS moraines lie far beyond the margins of the more recent ice advances despite larger global ice volumes during recent glacial cycles (*42*). The leading “regolith hypothesis” for this transition is that erosion of sedimentary cover overlying the Canadian Shield converted a soft, deformable bed that allowed the LIS to expand laterally via till deformation into a rigid one that supported a thicker and less laterally extensive ice sheet (*10*). Old moraines from mountain glaciers similarly lie far beyond the extent of more recent glacial advances. Here, glacial erosion of deep valleys forced subsequent ice advances into lower altitudes and warmer temperatures, limiting glacier length (*13*). Assuming a temperature lapse rate of 5.1°C/km (*43*), 71 m of mean erosion would increase temperature of an equally thick ice sheet by 0.4°C. Erosion of the deepest troughs would increase the temperature experienced by the ice sheet by 3° to 5°C and generate calving margins where the ice sheet scoured below sea level (*37*) or formed closed depressions that filled to become lakes (*41*). Discharge to outlet glaciers flowing through these deep scours thinned the LIS interior (*44*), giving them outsized importance to ice-sheet mass balance. Our quantification of subglacial erosion therefore indicates that the landscape-evolution feedback may also decrease ice-sheet extents through time for a given climate forcing, even in low-relief environments. Such topographic change may therefore explain some of the reduced areal extent of Middle-to-Late Pleistocene glacial advances, although the counterintuitive increases in ice volume still require consideration of basal friction (*10*) and the periodicity of climate forcing. Reduced ice volumes at the southern LIS margin could be offset by increases along the western LIS margin, where empirical data (*14*) and modeled ice-sheet reconstructions (*45*) indicate an expansion of the LIS at higher latitudes during the LGM.

Landscape and drainage change have remained major open questions in the geologic history of mid-continental North America. By reconstructing spatially distributed erosion and deposition, we demonstrate that an exponential fingerprint of glacial erosion persists from the bedform (*30*) to the landscape scale. Furthermore, the mapped glacial erosion suggests that continental glaciers demonstrate self-limiting behavior (*9*) by “digging their own graves” (*13*) and that this may complement erosion to rigid bedrock (*10*) in explaining smaller ice-sheet extents despite more extreme glacial cycles after the mid-Pleistocene transition. Our reconstructed end-Pliocene surface likewise provides the necessary starting point to test feedbacks between landscape evolution and ice-sheet dynamics (*23*, *46*), including the regolith hypothesis (*10*) for the mid-Pleistocene transition, and the accuracy of glacial erosion laws (*24*, *47*). Last, our map of the Pliocene landscape and its rivers is the first glimpse of the quantitatively reconstructed topographic surface of North America before glaciation. Combining this with maps of the bedrock and modern land surface illuminates the power of ice in reshaping topography.

## MATERIALS AND METHODS

To create a bedrock-surface topographic map, we compiled various high-resolution (typically 1:500,000 scale or better) state and provincial maps. These disparate datasets of buried bedrock surfaces (figs. S1 and S2) are available in either digital or analog map formats from most states and provinces within the Laurentide region. We applied three approaches to convert multiple bedrock-elevation data sources to elevation contours in digital vector formats for use in a surface interpolation algorithm and summarize these methods in fig. S1. (i) In the “direct-use” approach, we directly ingested and integrated existing digital contours and bedrock DEMs that we converted to digital contours. (ii) Using a “refinement” approach, we integrated local data with edge-matching datasets (e.g., at the Canada/U.S. border) to align valley and ridge features and/or augment base datasets with additional information from published work. An example of this refinement step was to incorporate Great Lakes bedrock contours from academic publications in place of the default lake bathymetry. (iii) Last, the absence of bedrock-elevation data in some areas required us to interpolate existing point bedrock elevations to produce bedrock-surface topographic contours at a resolution consistent with our larger-scale dataset. We obtained the depth of the bedrock interface from drilling logs and prepared data for interpolation (*48*), including filtering problematic data because of incorrect locations, geologic materials without depth assignments, and/or inaccurate ground-surface elevations.

We generated the complete bedrock surface by assimilating different elevation data types, such as points and polylines (contours), from the sources described above. To do this, we used the ANUDEM gridding algorithm (*49*–*51*), which applies a computationally efficient, iterative finite difference interpolation approach that maintains surface continuity using a thin-plate spline. It minimizes a terrain/user-specified roughness penalty while also using a conservative drainage enforcement algorithm that removes sinks while not imposing drainage conditions that contradict elevation data (*49*). The algorithm also minimizes a weighted sum of squared residuals from the input elevation data and the resulting surface grid (*49*). We statistically validated the final surface interpolation using borehole data from eight states and provinces where approximately 1 million data points (*N* = 938,157) were used to obtain an average root mean square error of 12.5 m (see Supplementary Methods and Materials and fig. S2).

To ensure that our preglacial topographic surface reconstruction is based on elevations that are not substantially affected by the ongoing transient solid-Earth response to deglaciation, we summed our bedrock-surface elevations with residual GIA, defined as the remaining amount of uplift and/or subsidence required to reach isostatic equilibrium. To do so, we used gridded model outputs from Raymo *et al.* (*19*), who simulated GIA using a variety of solid-Earth rheologies. From their multiple model runs, we chose outputs with a 71-km elastic lithosphere and upper- and lower-mantle viscosities of 3 × 10^20^ and 3 × 10^21^ Pa s, respectively. The 71-km lithospheric elastic thickness is consistent with independent elastic-thickness estimates for the southern Laurentide region of North America (*52*, *53*). This elastic thickness falls between the 90-km value used in VM2 for coupling with ICE-5G (*54*), the ice-thickness reconstruction used by Raymo *et al.* (*19*), and the 60-km value used in the newer VM5a model (*55*), designed to better fit GIA observations in the area south of the Last Glacial Maximum LIS, which our study area includes. Our mantle viscosity values are similar to those from the GIA-calibrated VM2 and VM5a mantle-rheology models (*56*). While consistent with VM2 and VM5a, this 10:1 ratio of lower:upper mantle viscosity is less than the 30-fold increase that would be consistent with observations of the long-wavelength geoid (*57*). We opted to more closely match VM2 and VM5a based on their joint calibration with ice-sheet reconstructions to simulate ice-age solid-Earth response to loading (*54*, *56*).

To construct the Pliocene surface, we first defined preglacial valleys and their gradients by following their centerlines and matching local channel elevations interpreted from geomorphic features and regional base-level indicators at the mouths of end-Pliocene watersheds. Bedrock valley cross sections (*N* = 187) were analyzed to define geomorphic features such as river terraces and truncated spurs (eroded ridgelines) and estimate local channel segment elevations. We interpolated channel elevations along with interfluve elevation constraints extracted from the bedrock surface DEM based on previous literature (*58*–*61*) describing preglacial uplands and undissected bedrock highs to create a continuous surface. This work involved three key assumptions. First, we assumed that regions of minimal glacial erosion are preserved as high anomalies in the bedrock topography. Second, we assumed that most tectonic uplift occurred southwest of the mid-continent (*5*, *62*) and that the southern Laurentide region was tectonically quiescent during the Pliocene. Third, we assumed that truncated spur ridges and corresponding strath terraces exposed in the surface topography and indicated in the buried bedrock topography represent relict floodplains and therefore the level to which the landscape was graded (figs. S3 to S6). Elevations along preglacial drainage divides and interfluves were conservatively approximated using bedrock highs buried in sediment-filled lowlands and exposed at the modern ground surface of unglaciated uplands (figs. S4 to S6). Last, we presumed that by linking these local indicators of preglacial channel elevations across a regionally reconstructed drainage, we could reconstruct a continuous Pliocene surface even though Quaternary landscape modifications occurred at different times and in different places.

On the basis of elevation constraints from the GIA-adjusted bedrock surface and paleodrainage patterns, we used ANUDEM (*49*–*51*) to reconstruct preglacial (i.e., end-Pliocene) topography. After creating the ANUDEM-generated interpolated surface, we registered it to the bedrock topography DEM and set all preglacial elevations calculated to be below the current bedrock surface to the present-day bedrock elevation. This final Pliocene surface formed the basis for our calculations of erosion during the Quaternary.

Watershed boundaries for five major preglacial drainages were extracted from the end-Pliocene DEM by establishing pour points near drainage outlets and applying standard flow-routing algorithms. We compared the DEM-derived watershed boundaries with the reconstructed mid-continental drainage (Fig. 2). If these watershed boundaries intersected the mapped courses of preglacial rivers courses, this would indicate that our chosen paleotopographic surfaces indicate a different preglacial drainage pattern. This could occur, for example, if we had picked a mix of features of different ages as our end-Pliocene (i.e., preglacial) land-surface control. The excellent match between our mapped preglacial river courses and the computed drainage basins from the end-Pliocene DEM confirms that the reconstructed drainage and base-level elevations distributed throughout coincide with a hydrologically correct digital elevation model even after incorporating full mantle relaxation (*19*) after the last glacial maximum.

We extracted river long profiles from the paleo-Saskatchewan, Missouri, St. Lawrence, Platte, and Wabash Rivers (Fig. 5). We flooded all depressions in the end-Pliocene DEM and then performed a D8 convergent flow routing across that surface. We defined each of these five reconstructed paleo-rivers to begin at headwaters with a threshold drainage area of 156,000 km^2^. This choice of threshold drainage area is purely for the purpose of extracting long profiles but is otherwise not meaningful because the headwaters for each of these five rivers extends beyond the boundaries of the study area. We then extracted the original (i.e., unfilled) end-Pliocene DEM elevations along each of these five rivers; extracting elevations from the unmodified DEM produces some of the irregularities visible in Fig. 5. These long profiles are largely concave-up, although the Missouri River and Wabash River display convexities associated with lithological change (both) and tectonic activity (Wabash), and the long profile of the Wabash begins downstream of its headwaters.

## Supplementary Materials

### This PDF file includes:

Supplementary Materials and Methods Figs. S1 to S10 References
