# Supplementary material for: Landscape evolution under the southern Laurentide Ice Sheet
Source: Sci Adv. 2021 Nov 24;7(48):eabj2938. doi: 10.1126/sciadv.abj2938 (PMC8612676; doi:10.1126/sciadv.abj2938)
Supplement: Supplementary file 1 — Supplementary Materials and Methods Figs. S1 to S10 References [file sciadv.abj2938_sm.pdf]

Science Advances
AAAS

Supplementary Materials for

Landscape evolution under the southern Laurentide Ice Sheet

Shawn Naylor*, Andrew D. Wickert, Douglas A. Edmonds, Brian J. Yanites

*Corresponding author. Email: snaylor@usgs.gov

Published 24 November 2021, *Sci. Adv.* **7**, eabj2938 (2021)
DOI: 10.1126/sciadv.abj2938

This PDF file includes:

Supplementary Materials and Methods
Figs. S1 to S10
References

Supplementary Materials and Methods

Mapping the bedrock surface

In the Great Lakes region, direct-use bedrock elevation data (BED, Fig. S1) were available for Indiana(*67*), Minnesota(*68*), northwestern Pennsylvania(*69*) and Lake Michigan(*70*). In Lake Michigan, depth data were converted to elevations in meters above mean sea level (masl) using published long-term average lake levels(*71*), and the lake bottom elevation was used as an upper limit on bedrock surface elevation.

Data refinement (Fig. S1) was applied to BED from Illinois, Ohio, Wisconsin, and Ontario. A bedrock map for Illinois was from existing data(*72*) and digitized analog maps in northwest Illinois(*73, 74*). Similarly, in Ohio, bedrock elevations came from the statewide compilation(*75*) and the Ohio Soils Survey Geographic Database(*76*). In Wisconsin, we converted 30-m contours of sediment thickness(*77*) into a bedrock surface by calculating an average thickness for each isopach polygon, rasterizing the data, and subtracting it from the ground surface DEM. Northern Ontario has <1m Quaternary sediment on the surface(*78*) so ground-surface elevations (Shuttle Radar Topography Mission data) were used for the bedrock surface. In Southern Ontario, we integrated bedrock surface and overburden thickness data(*48*) with contours from two bedrock surface maps(*79, 80*).

Data refinement processes (Fig. S1) were applied to BED from Lake Erie, Lake Huron, Lake Ontario, and Lake Superior due to thick lake floor sediments. In Lake Ontario, the bedrock surface was mapped from seismic data(*81*) and we digitized the analog map contours. In Lake Huron, bathymetry contours(*82*) were edited to be consistent with features in Georgian Bay that show a bedrock ramp at Wasaga Beach, Ontario(*83*). Contours near Port Huron, MI were modified to match Southern Ontario on-shore bedrock contours(*48*) and Michigan water well records that place the bedrock surface along the southern end of the lake in the 150-160 masl range indicating thick littoral deposits above bedrock. Contours in Saginaw Bay were edited to match the base of glacial sediments at 162 masl(*84*). In Lake Erie, BED comes from published data for the northern part(*48*), and bathymetric contours(*85*) for the southern part. In the Lake Superior basin, bedrock elevations come from seismic reflection profiles(*86*) that were digitized and then interpolated. We edited Lake Superior bedrock elevations based on preliminary data(*87*) showing deeper north-south trending valleys in the eastern lake basins.

Michigan bedrock surface data compiled as part of a state-wide mapping effort(*88*) were provided by personnel at Michigan State University. Data refinement included contouring the DEM and edge-matching data boundaries along the Lake Superior shore of the Upper Peninsula, as well as the intersection between the southern edge of the Lower Peninsula and Indiana.

In Northern Kentucky, we created a bedrock surface from water-well logs(*89*). A statistical analysis of this interpolation is provided in the Technical Validation section, below.

In the Interior Plains region, Direct-use BED were available for Iowa(*90*), Alberta(*91*), South Dakota(*92*), Saskatchewan(*93*), and Nebraska. The BED in eastern Nebraska are from aquifer base elevations that originate from digitized bedrock contours(*94-96*).

We compiled and refined bedrock surface data for northwestern Missouri, Kansas, Montana, and North Dakota. We digitized and edge-matched existing bedrock surface maps for northeastern Montana, southeastern Saskatchewan, and northwestern North Dakota(*97*). We georeferenced and digitized previously published bedrock contours for the rest of North Dakota(*98*). Similarly, existing bedrock surface maps of northeastern Kansas(*99*) and northwestern Missouri(*100*) were digitized, georeferenced, and attributed with elevation values. In northeastern Missouri, we interpolated a bedrock surface from water-well logs with bedrock contact designations(*101*).

For Manitoba, the existing bedrock surface map(*102*) contains sparse data along the Pembina Escarpment (G. Keller, personal communication, 17 December, 2015), therefore, we digitized and incorporated previously mapped bedrock contours(*103*). Additionally, eastern Manitoba is mapped as bedrock outcrop(*104*) and we used ground-surface elevations (Shuttle Radar Topography Mission data) for the bedrock surface in this region.

Bedrock surface resolution

We interpolated the final bedrock surface interpolation to 250-m raster cell resolution. We chose this resolution because a 1:500,000 map scale corresponds this raster cell resolution(*105*) and the source data were predominantly mapped at the 1:500,000 scale or better. We compared the final DEM to a 250-m resample ground surface DEM derived from 1-arc second Shuttle Radar Topography Mission (SRTM) data(*106*), and cells with bedrock elevations above the ground surface were set to equal the SRTM elevation.

Technical validation of the bedrock surface

We conducted a spatial statistical analysis to ensure that the final ANUDEM interpolation results, derived primarily from digital contour input data, compare well with point data throughout the study area. Geologic boring records with bedrock elevations were readily available from 8 states and provinces (Indiana, northern Kentucky, Manitoba, Michigan's Lower Peninsula, Minnesota, northeastern Missouri, Ohio, and southern Ontario) to conduct a statistical analysis of the final 250-m bedrock-surface topography DEM. The assessment points were paired with elevation values from the final interpolated DEM at the same locations. An ordinary least squares regression analysis indicated regression coefficients (R) between 0.95 and 0.99 for each of the analysis regions (Fig. S2). We also calculated root mean square error (RMSE) between ground-truth point data and the associated raster elevation cell in each of the state/provinces (Fig. S2) and obtained a mean RMSE of 12.5 m and a range from 8 to 17 m of RMSE values for individual states/provinces. This is close to the range of error in the SRTM specification (16 m), and error between the gridded data set and point measurements can also be produced by sub-grid topographic variability in bedrock surface elevation.

Residual glacial isostatic adjustment (GIA)

The GIA correction produced an additional 3 to 96 meters of uplift, which we applied to the modern bedrock surface. The greatest amount of residual uplift lies in the northeastern portion of the study region, which hosted thicker ice that also retreated most recently. Because of the radial pattern of GIA around the former Laurentide Ice Sheet and increasing residual GIA magnitudes farther within its footprint, the Paleo-Missouri River and Paleo-Saskatchewan River long profiles (see below) were most strongly impacted by this residual GIA.

Erosional isostatic adjustment was not simulated because (1) insufficient data exist beyond the study area to constrain the spatial distribution of erosional unloading and (2) magnitudes of expected erosional isostatic deformation (~100 m) are similar to uncertainties among different tuned solid-Earth models for dynamic topography in Canada(*56*). Although we constrain mass redistribution within the study area, erosional isostatic response is impacted significantly by mass redistribution within one flexural wavelength (~500–1000 km) of the study region margins. Furthermore, local isostatic adjustment links solid-Earth rheology with both surface and subsurface loads, and as such, significant research on the deformation of mid-continental North America must be completed in order to solve this joint problem. We acknowledge that the isostatic effects of Quaternary erosion, as well as long-wavelength land-surface deformation due to dynamic topography, represent an important problem for future research. Therefore, we created an extensive data package from this work to support future research into surface deformation. Our geologically constrained river courses and paleotopography, alongside changes in surface loading from our reconstructed erosion and deposition depths, should help to limit and inform plausible geophysical solutions.

<u>Strath terrace elevations and pre-glacial (end-Pliocene) surface reconstruction</u>
Multiple strath terrace levels are present in the modern Missouri River basin(*107*) and in the buried bedrock topography of the Southern Paleozoic Basins and Arches province (Fig. 1) as indicated by "deep stage" bedrock incision (i.e., incision to the deepest point along a buried-valley cross-sectional profile) events(*65, 108*). The occurrence of multiple terrace levels that record former floodplain elevations is problematic when establishing consistent paleo channel elevation indicators along a valley. We overcome this issue by generally choosing prominent valley-bounding strath terraces (Fig. S6) and ensuring that the elevations along a channel coincide with a consistent gradient as shown by the sequence of profiles interpreted in Figures S5 and S6. Furthermore, we integrate data from previous studies on Late Neogene geology when available such as the Flaxville and Souris River stratigraphic units(*109*) present along the section of the paleo Missouri River shown in Fig. S5. The robustness of this method is further supported by pre-glacial river longitudinal profiles (Fig. 5).

<u>Reconstructing pre-glacial channel and interfluve patterns</u>
Paleo Saskatchewan and Missouri Rivers
The west-to-east orientation of valleys that routed eastward drainage across the Pliocene Canadian Plains(*110, 111*) is reconstructed using low-gradient channels delineated from strath terraces along major valleys in the Rocky Mountain foothills that trend toward eastern modern-day channels feeding Hudson Bay (Figs. 2, and S5). The northernmost valley in the Paleo Saskatchewan drainage network follows the trend of the modern Beaver River course and has headwaters consistent with those of the modern Peace River (Fig. S10). Others have documented the historic path of the Upper Peace River toward Hudson Bay(*112*). To the south, the current Upper Missouri River and the ancestral South Saskatchewan River(*113*) also flowed toward Hudson Bay. These reconstructions are consistent with paleoflow indicators in the Cenozoic gravels of Western Canada and the northern United States(*109*). Pre-glacial elevations in northern North Dakota were generally consistent with the modern Missouri Escarpment(*36*). Similarly, geomorphic analyses of terraces in the U.S. Black Hills(*107*) as well as Late Miocene fluvial benches of the northern U.S. and western Canada(*109*) provide guidance for inferring pre-glacial floodplain elevations and river longitudinal profiles in the region.

Paleo St. Lawrence River

Early work in the Great Lakes led to a proposed pre-glacial drainage that followed the axes of the lakes except where the pre-glacial "Laurentian River" crosscut southern Ontario between the Georgian Bay of Lake Huron and Toronto, along the northwestern shore of Lake Ontario(*114*). This interpretation is largely supported by later studies investigating the lithologic (differential erodibility of carbonate and shale rock units) and structural (bedrock jointing and rock unit dip) controls on Great Lakes Region bedrock valley orientation(*114, 115*) and regional bedrock-surface morphology(*60, 83*). More recently work by Carson et al.(*1*) provided evidence for the pre-glacial "Wyalusing River" with headwaters near the modern confluence of the Mississippi and Wisconsin Rivers in the mid-continent and which flowed northeastward towards the Lake Michigan basin. The Sawtooth Mountains to the north and west of Lake Superior are interpreted as the northern pre-glacial divide of the Paleo St. Lawrence River drainage as are bedrock highs north of Lakes Michigan and Huron. Pre-glacial tributaries in the southern portion of the St. Lawrence drainage, were sourced from topographic highs along a cuesta formed by resistant Paleozoic strata between Lake Michigan and Lake Erie (Fig. 1). Here, we used high bedrock surface elevations to interpolate a divide between this drainage and the ancestral Wabash River to the south.

Paleo Wabash (Teays) River

Adjacent to the Great Lakes, the current southwesterly path of the Ohio River toward the Mississippi River is widely known to be a result of Quaternary glacial diversion(*59, 65, 116-120*), but its pre-glacial course is debated. Some researchers identify the Teays-Mahomet buried valley system, spanning from West Virginia in the east to Illinois in the west, as the pre-glacial drainage(*120*). Others suggest that Pliocene rivers east of Indiana flowed to the north into the Lake Erie basin(*59*), formerly occupied by the Erigan River(*39, 121*). However, most researchers acknowledge the northwest trending entrenched Teays Valley in Ohio as the pre-glacial main channel that drained much of Ohio and northern Kentucky(*108*).

Preserved sediments and landforms provide evidence that the Old Kentucky and Licking Rivers in northern Kentucky (Fig. S10) once were the headwaters of a northwestwardly flowing system that was abandoned during the Quaternary diversion/capture of the modern Ohio River(*117, 119*). Valley morphology and abandoned terraces capped with gravel deposits indicate that the valley containing the present-day South Fork of the Licking River once held the pre-glacial trunk stream(*119*); this course lines up well with the Whitewater River Valley in southeastern Indiana. Both the Whitewater River Valley and the valley of the South Fork of the Licking River follow the lithostructural margin of the Paleozoic Illinois basin. The buried bedrock valley below the Whitewater River connects with the buried Anderson Valley to the north(*67*) (Fig. S3) and eventually to the ancestral Wabash River that flowed toward the Gulf of Mexico. Elevations of the previously mapped high-level pre-Quaternary gravel deposits in the Licking River Valley(*119*) provide pre-glacial channel and floodplain elevations along the southern headwaters of the ancestral Wabash River. Similarly, field investigations in the Hocking River Valley of southeastern Ohio(*122*) indicate that early- to mid-Quaternary deposits exist along the current drainage divides of high-order watersheds suggesting that pre-glacial headwater channel elevations were closer to current ridgeline elevations in the region. This is consistent with slackwater sediments on terraces associated with the northward flowing ancient Pittsburgh River

in western Pennsylvania and northeastern Ohio(*123*). These high-level terraces exist at ~330 m and were dated as early Quaternary (prior to 0.774 Myr BP; millions of years before present) based on their reversed magnetic polarity(*123, 124*). Headwater channel elevations for the shortened ancestral Ohio River are estimated based on strath terraces near the pre-glacial drainage divide for the ancestral Ohio River basin near Madison, Indiana, where an undissected upland exists(*120*). The elevations are consistent with cosmogenic dating work in the Ohio River basin that suggest Quaternary incision rates of ~30 m/Myr, mostly during three primary pulses of river downcutting(*125, 126*).

In the modern Wabash and Ohio River basins (Figs. S4 and S10), bedrock strath terrace elevations visible in the bedrock topography (Fig. 1) do not clearly support the previously suggested east-west course of the pre-glacial Teays that drained into the modern Mississippi channel. The interpretation presented herein, based on 180-215-m strath elevations in the central Paleo Wabash valley and the longstanding tectonic activity in the Wabash Valley Seismic Zone(*127*), is that the upper Teays was a tributary to the Paleo Wabash River and not to the modern Mississippi channel.

Paleo Platte River
In the western portion of the current Mississippi River basin, the present-day Lower Missouri River developed as an ice-marginal feature(*128*) and the paleo Platte River was the major Pliocene drainage to carry water from Nebraska, northern Kansas, and northern Missouri(*5*). Calculated post-depositional tilt for the Miocene-Pliocene Ogallala Group based on the preserved channel-scour geometry and grain size(*62*) indicates that the eastern portion of the Paleo Platte, which lies in our study area, did not experience uplift. Therefore, SRTM elevations of Cenozoic sediments exposed and mapped at the ground surface in central Nebraska are used to represent the trend of pre-glacial topography in the headwaters of the Paleo Platte. The bedrock surface data presented (Fig. 1) indicates that the Paleo Platte River drained a large portion of the mid-continent west of the Indiana and Illinois state border (Fig. S3).

Geologic mapping indicates that the precursor to the central Mississippi River course is the ancestral Illinois River(*129*), a southward flowing tributary of the Paleo Platte River (Fig. 2). North of this, bedrock elevation data indicate that the northern portion of the modern upper Mississippi River system drained towards the Paleo St. Lawrence River(*1*). Near the headwaters of the ancestral Illinois River, regionally correlated geomorphic surfaces consistent with the pre-0.774 ka Bridgeport strath terrace(*58, 124, 130*) along the pre-glacial Wyalusing River(*1*) provide minimum elevation constraints on the pre-glacial Gulf of Mexico / St. Lawrence River drainage divide (Fig. S9). The reconstructed position of this pre-glacial continental drainage divide as well as its northwards relocation during the Quaternary are consistent with Pliocene drainage-basin reconstructions based on the sedimentary record in the Mississippi embayment, which that indicate a major Pliocene-Quaternary reorganization of the Missouri River(*5*).

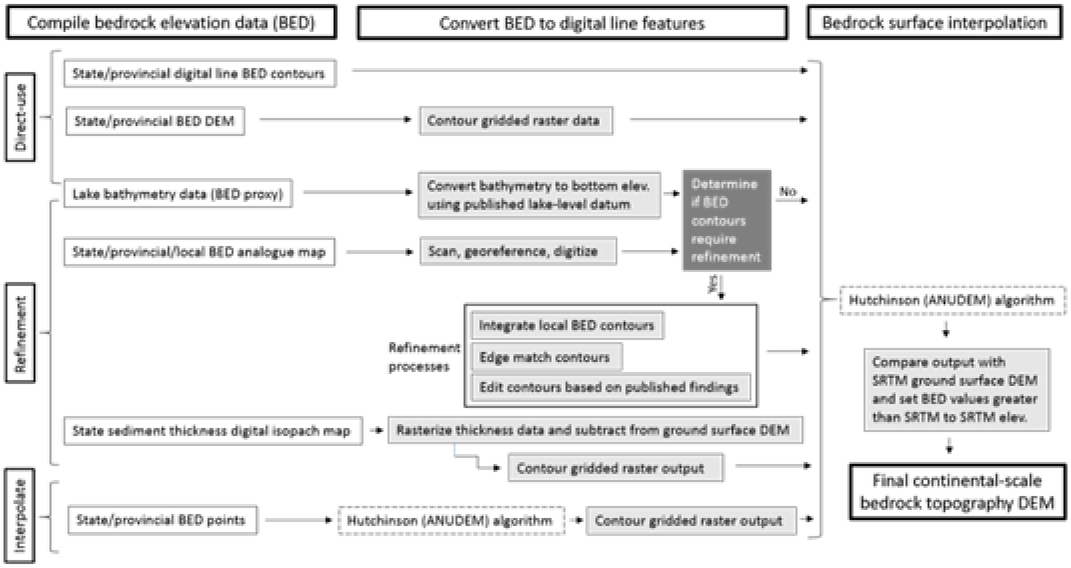

Fig. S1. Diagram showing the bedrock elevation data compilation, data processing, and bedrock surface interpolation approach.

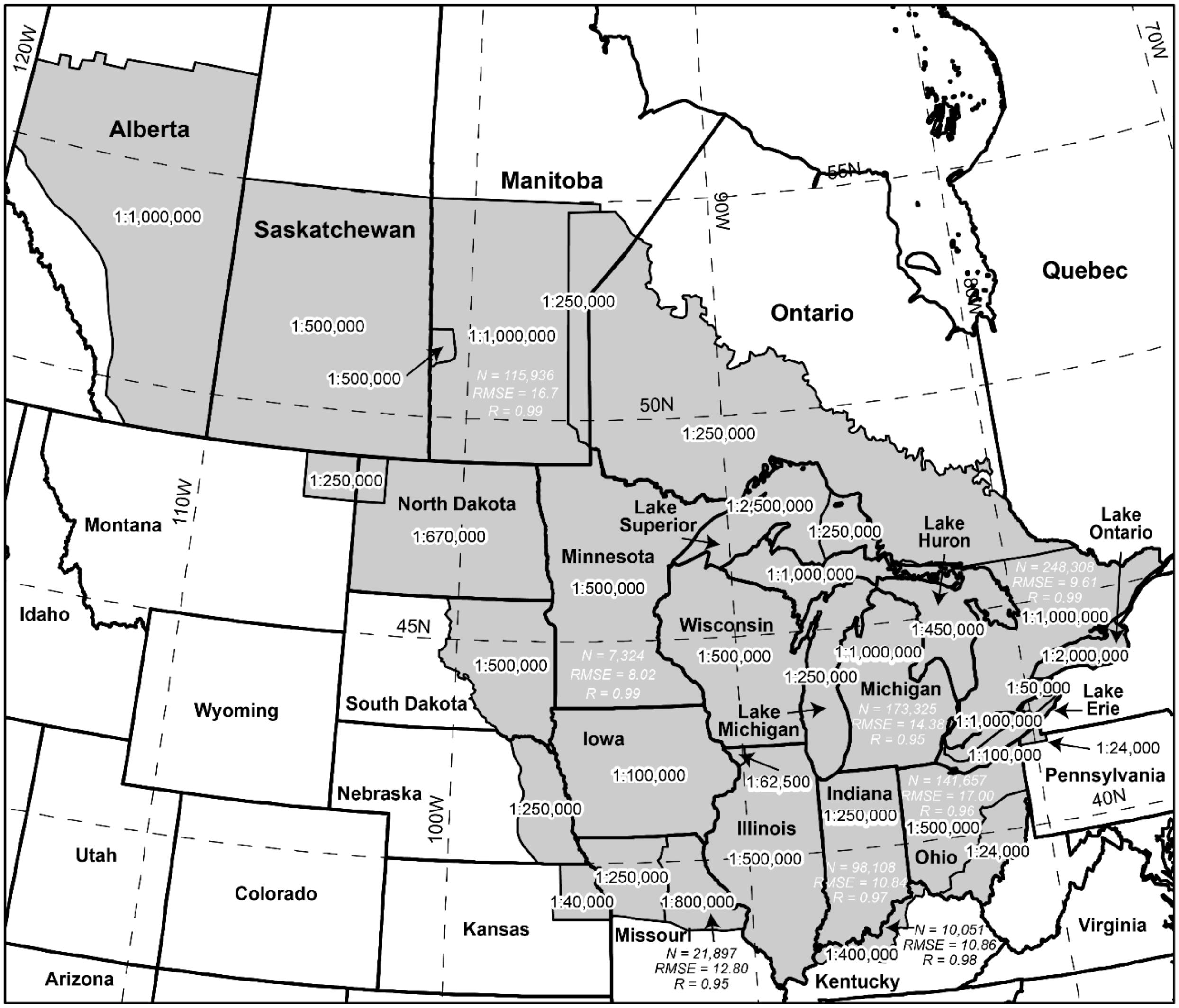

Fig. S2. Bedrock elevation data (grey) compiled for the Laurentide region.
Map scales for gridded data are approximated using Tobler's Rule. Sample number (N), root mean square error (RMSE) and regression coefficient (*R*) are based on a statistical analysis of the final interpolated bedrock surface and available bedrock elevation point data discussed in the Technical Validation section.

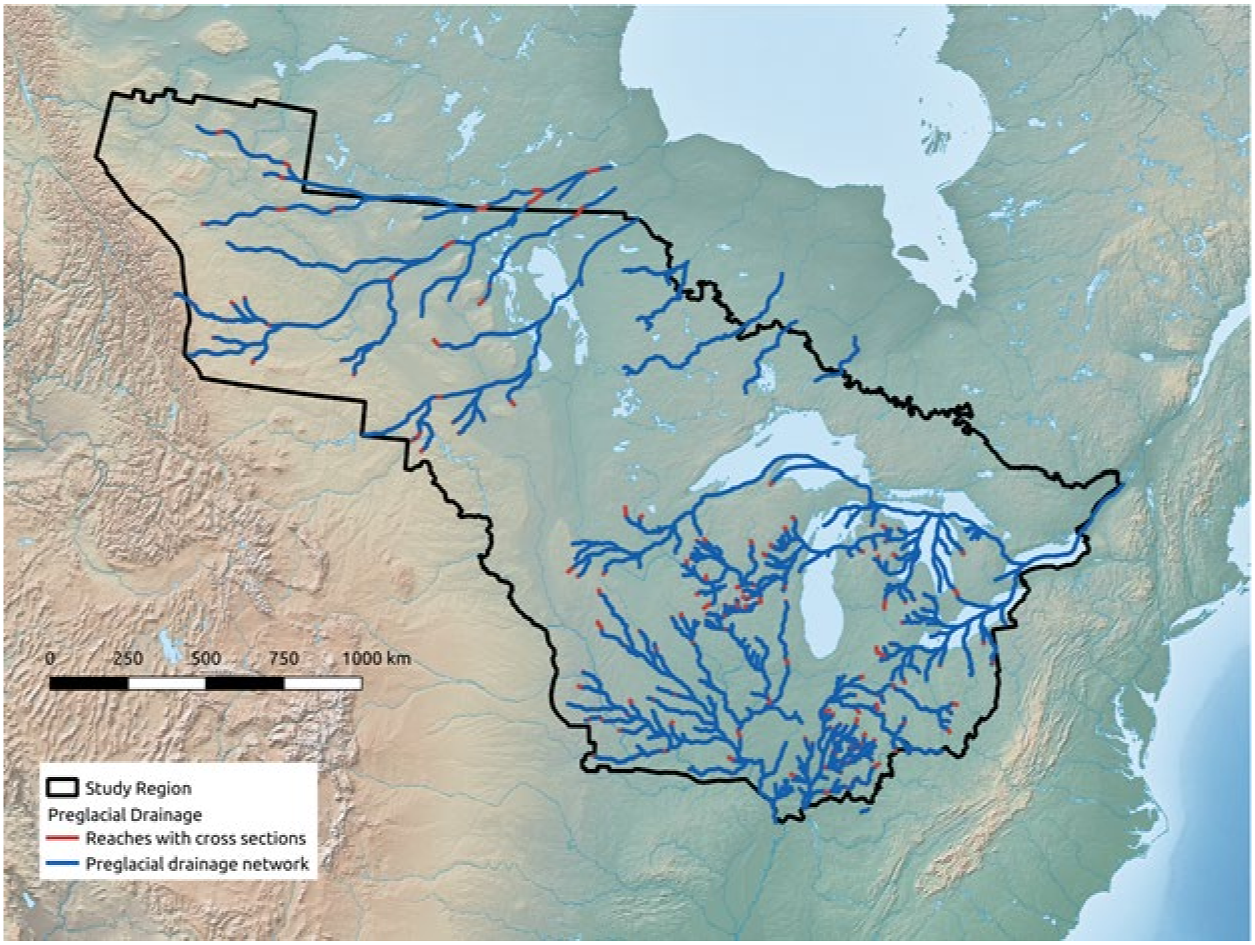

Fig. S3. Mapped preglacial drainage and strath locations.
These drainage patterns, based on buried valleys and geological relationships (see Methods), helped to define the structure of the Pliocene surface using ANUDEM. The orange reaches denote regions in which we constructed valley cross sections and mapped straths (Figs. S4–S6) to further constrain paleotopography.

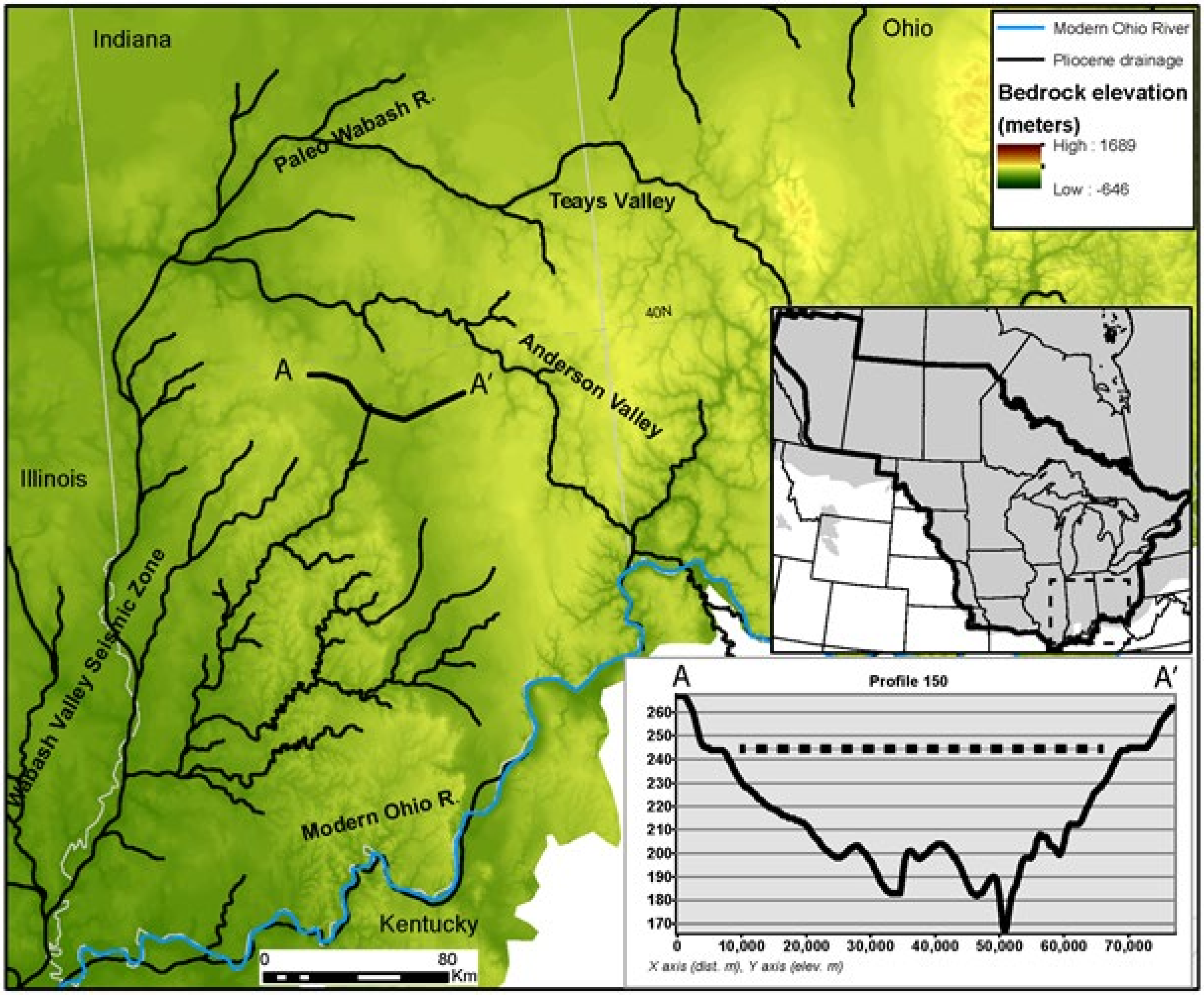

Fig. S4. Example profile (A to A') of bedrock strath terrace used to develop the end-Pliocene surface in the Paleo Wabash (Teays) drainage.

The dashed box on the inset map (same inset map from Fig. 1 of the main text) shows the location of this region relative to the full analysis extent. The dashed line in the valley profile indicates the interpreted elevation of the strath terrace, and this elevation was used when interpolating the Pliocene topographic surface at the channel reach location.

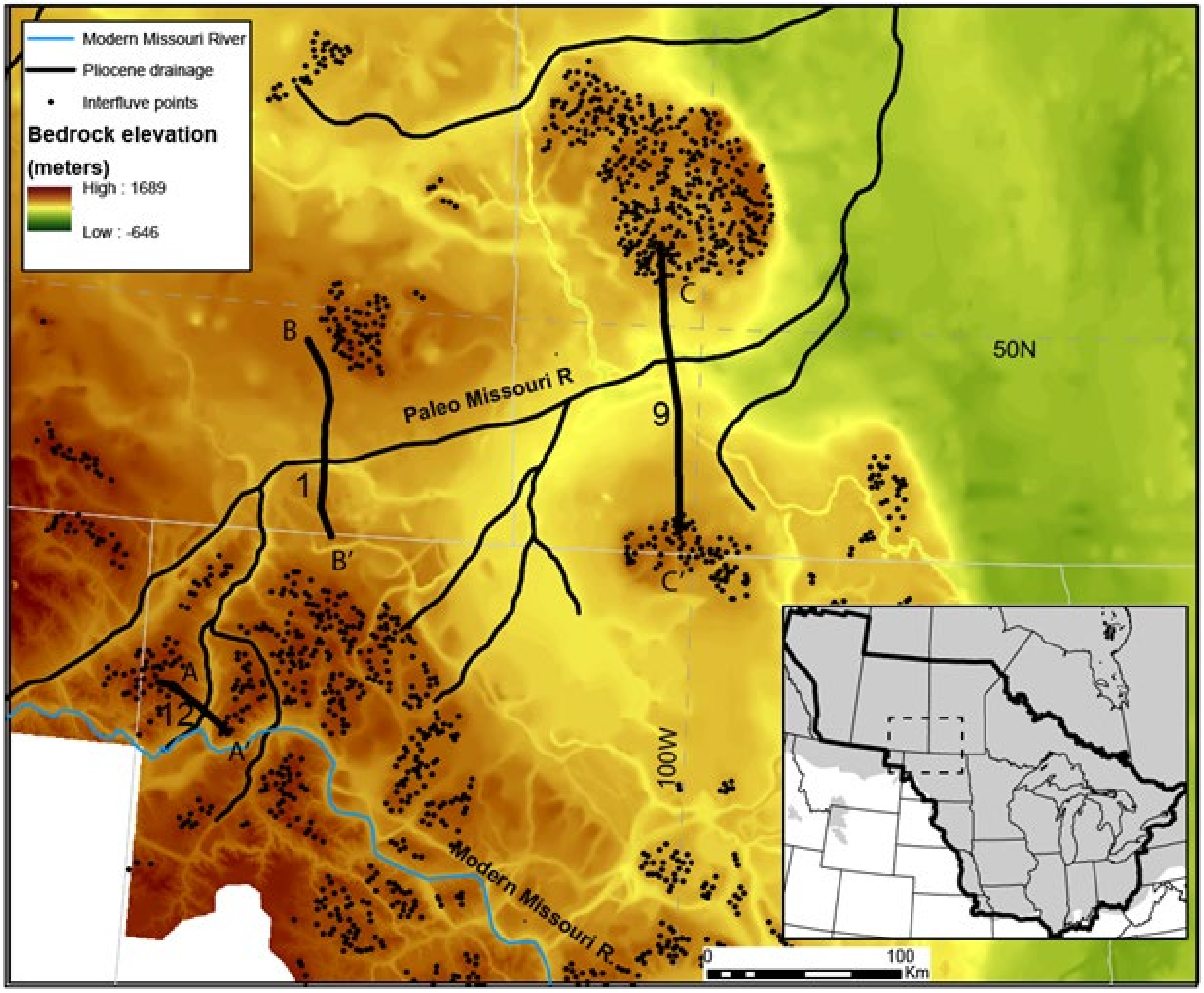

Fig. S5. Bedrock topography of the Western Plains near the Canada – United States border and locations of valley profiles used to interpret end-Pliocene strath terrace elevations. The dashed box on the inset map (same inset map from Fig. 1 of the main text) shows the location of this region relative to the full analysis extent. The gradient along the Paleo Missouri River main channel is established using strath elevations such as those shown in Fig. S6. Buried valleys cross-cut one another and modern river courses such as the current Missouri River valley.

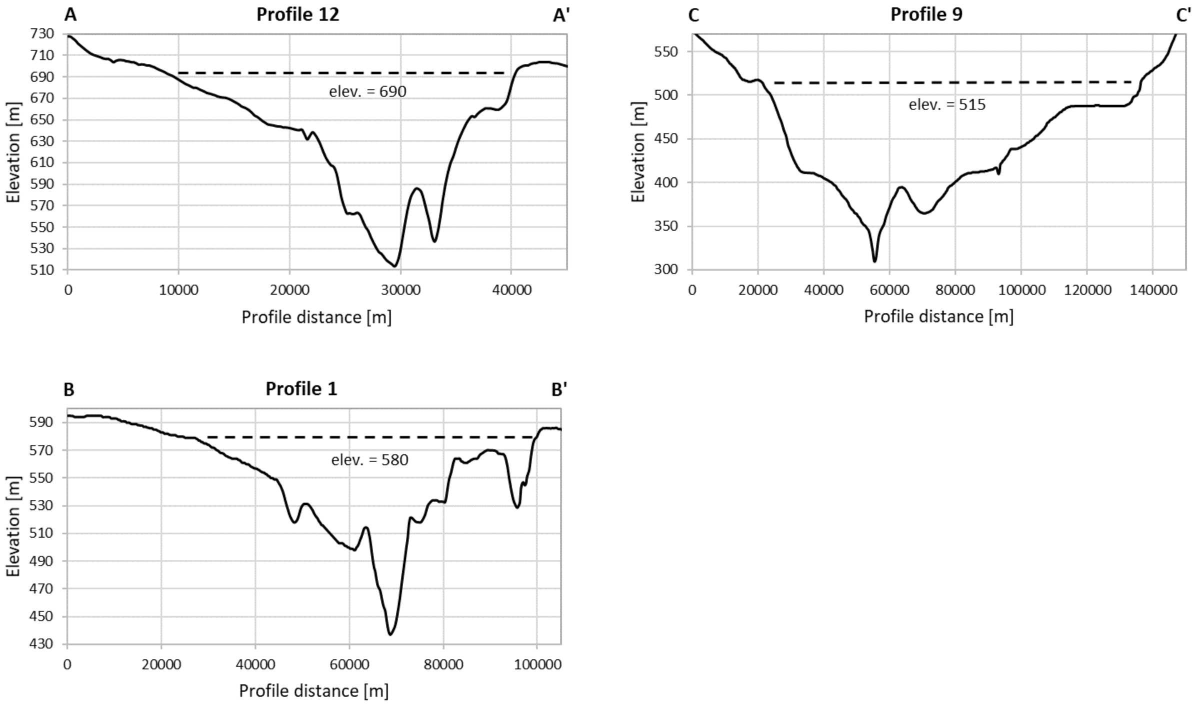

Fig. S6. Valley profiles along the Paleo Missouri River showing how valley-bounding strath terraces are selected when multiple benches are present along a valley profile.
The profile locations are shown in Fig. S5.

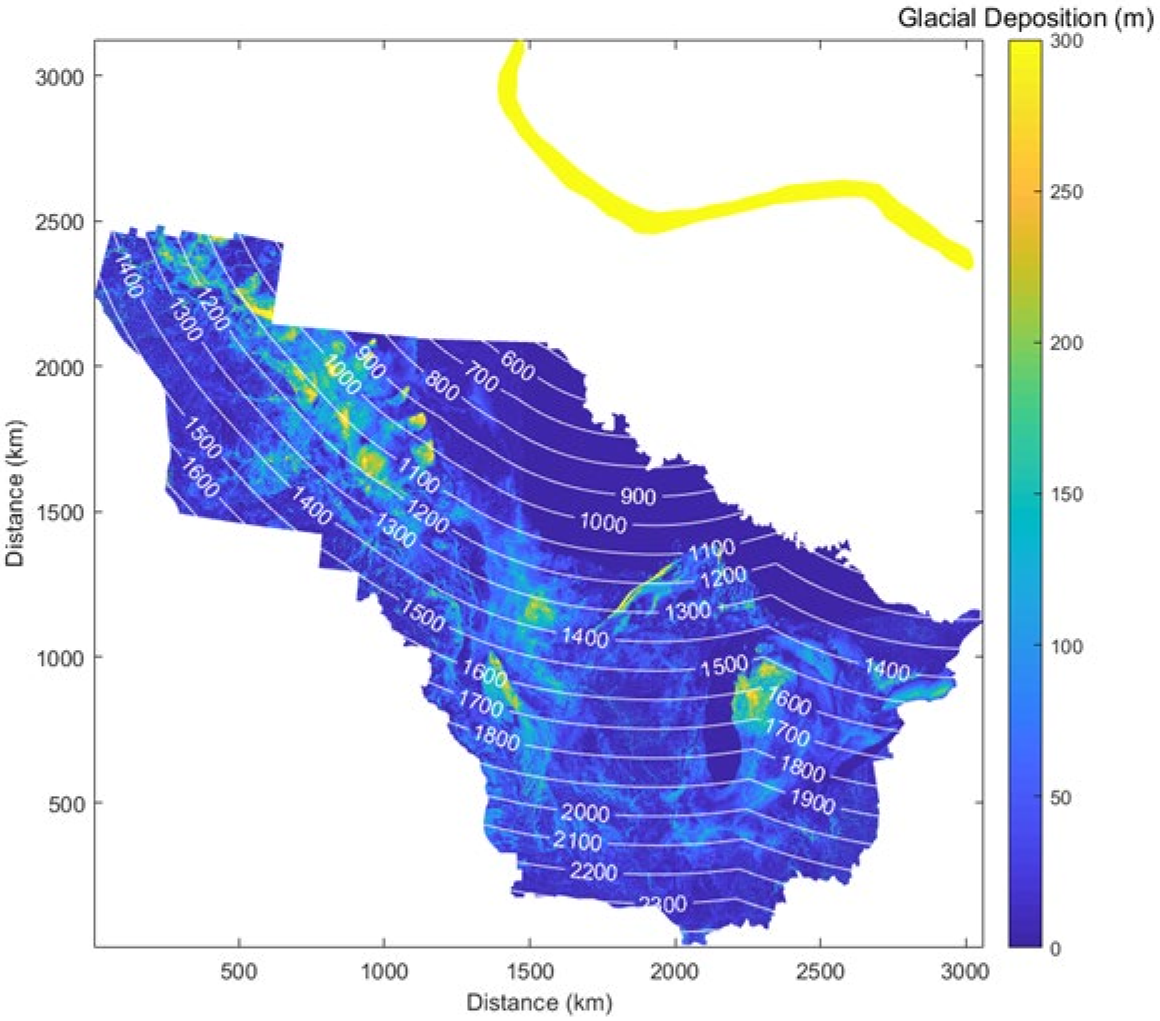

Fig. S7. Glacial sediment deposition with contour lines showing distance (km) from the Keewatin and Quebec-Labrador ice domes and connecting ice saddle (yellow area to the north of study area) derived from sediment dispersal patterns(*21*).
Colorbar is glacial sediment deposition in meters.

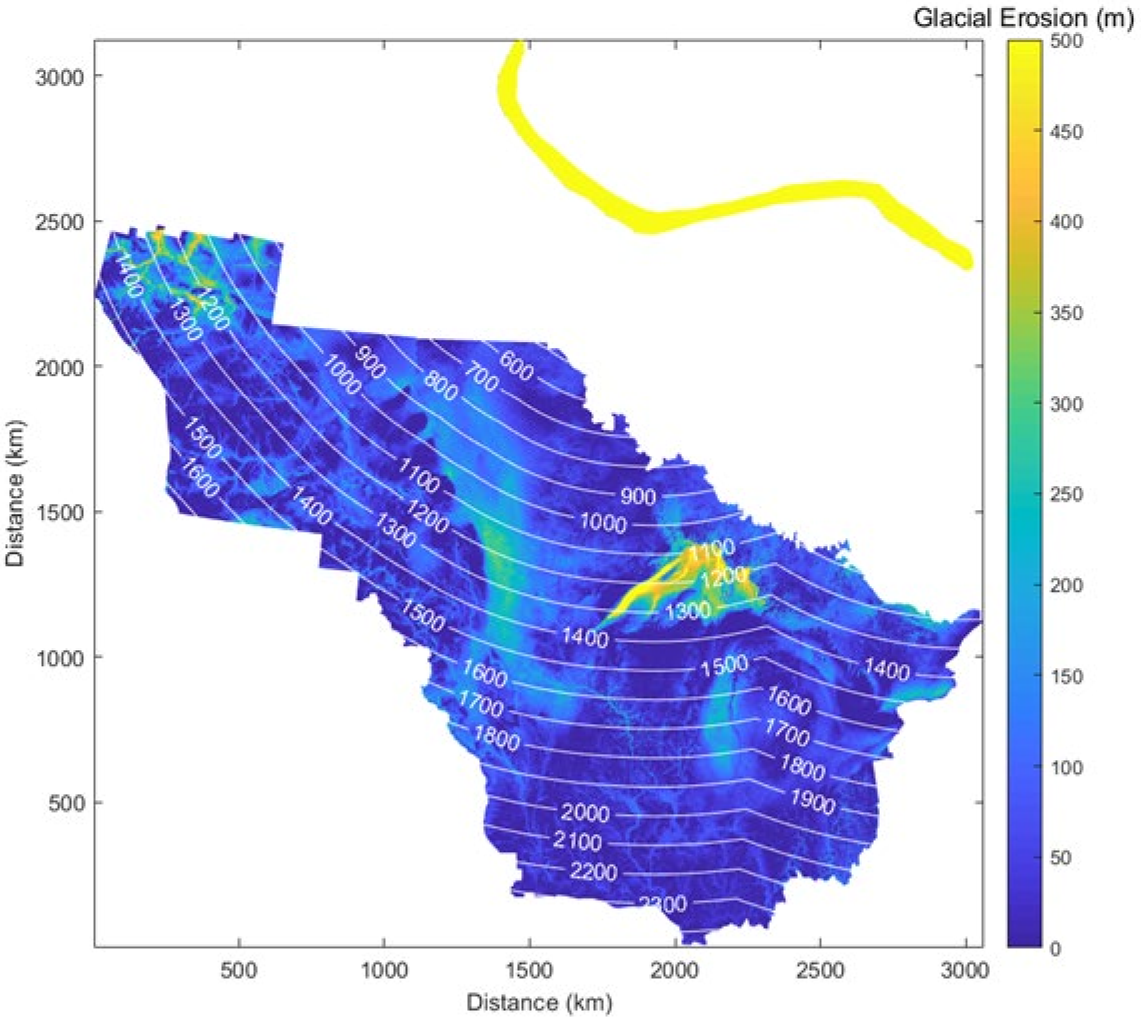

Fig. S8. Bedrock erosion with contour lines showing distance (km) from the Keewatin and Quebec-Labrador ice domes and connecting ice saddle (yellow area to the north of study area) derived from sediment dispersal patterns(*21*).
Colorbar is glacial sediment erosion in meters. The scale of the colorbar is limited to 500 m but there are erosion extremes that reach 1000 km in the Lake Superior basin (location shown in Fig. 1).

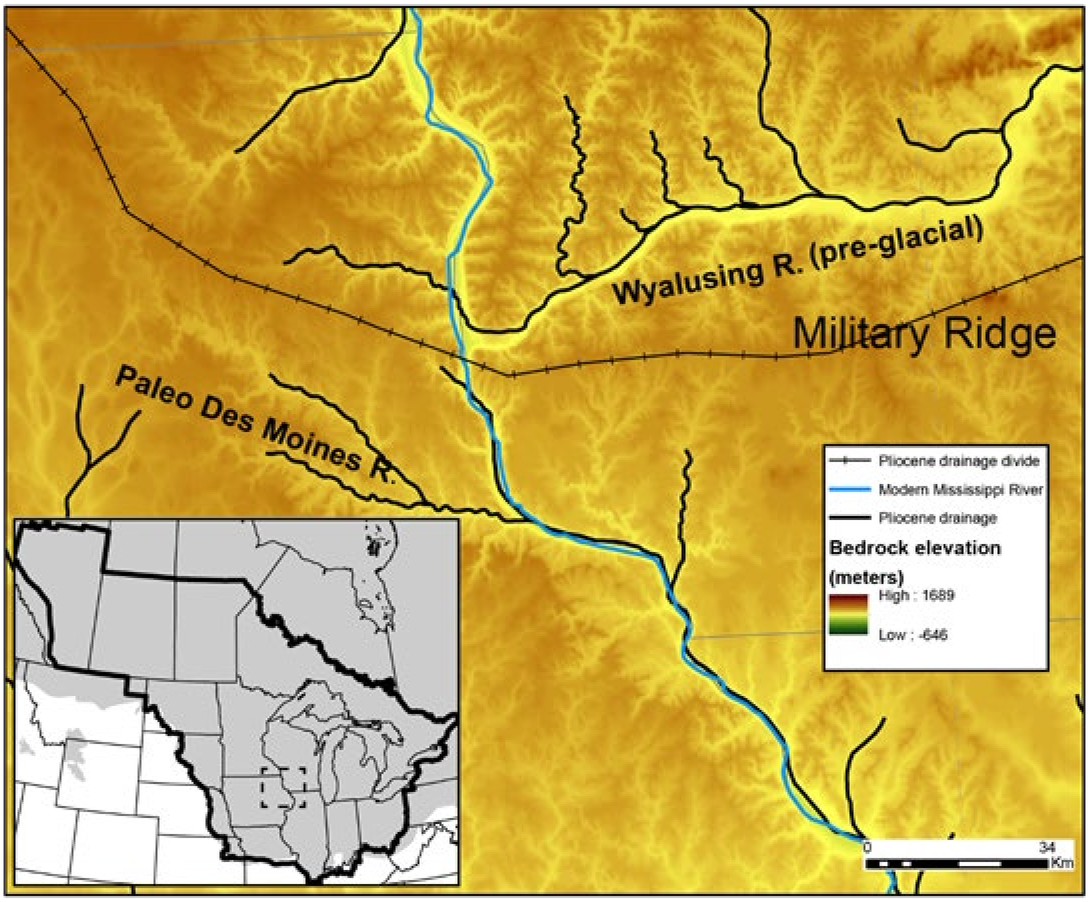

Fig. S9 Mississippi River valley bedrock topography showing diversion from end Pliocene drainage.

The dashed box on the inset map (same inset map from Fig. 1 of the main text) shows the location of this region relative to the full analysis extent. The Wyalusing River was dammed during an early Quaternary LIS advance(*6*) and flow was diverted through Military Ridge(*1*) to create the incised modern Mississippi River channel.

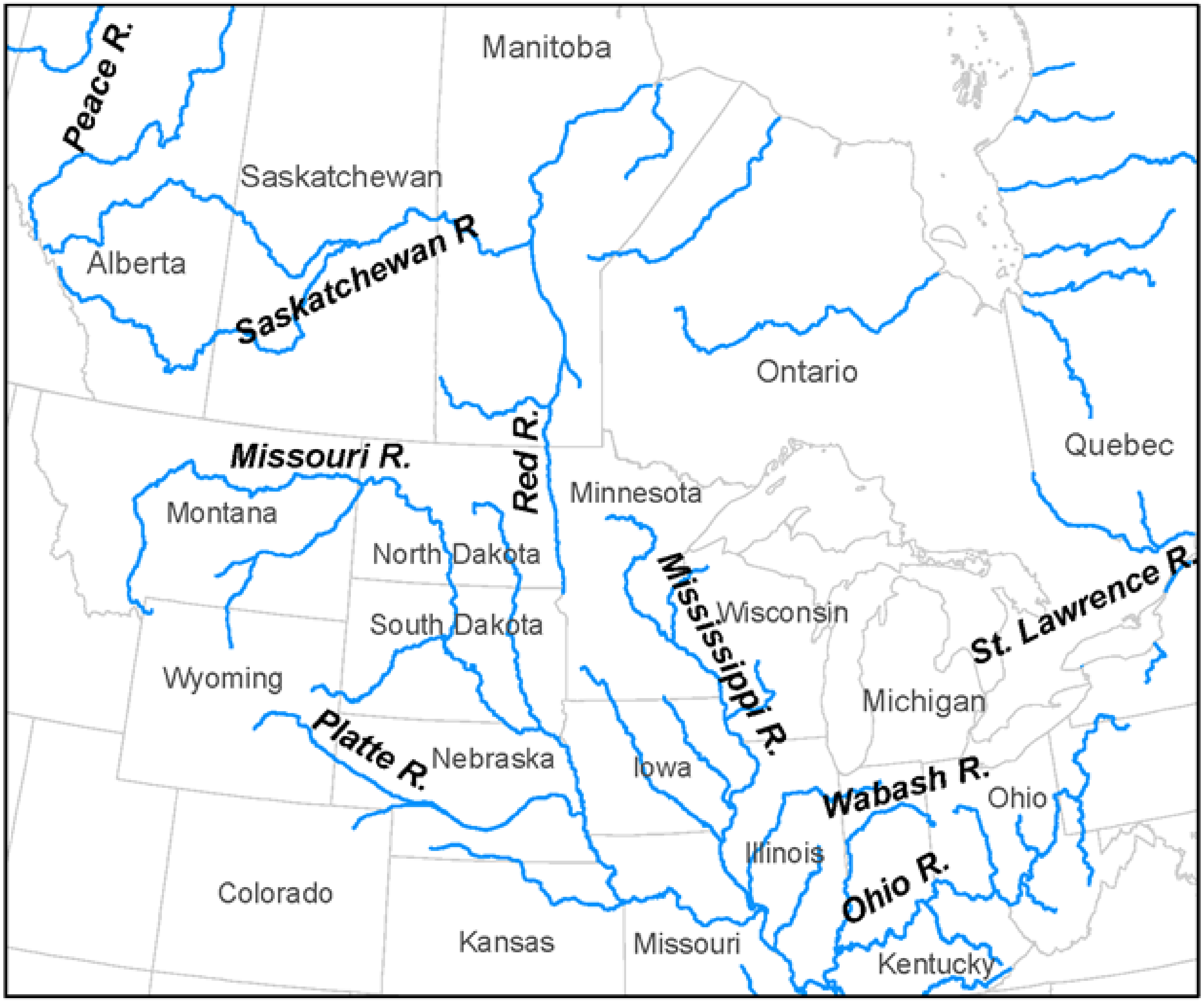

Fig. S10 Modern configuration of major rivers in central North America.

REFERENCES AND NOTES

1. E. C. Carson, J. E. Rawling III, J. W. Attig, B. R. Bates, Late cenozoic evolution of the upper Mississippi river, stream piracy, and reorganization of North American mid-continent drainage systems. *Geol. Soc. Am.* **28**, 4–11 (2018).

2. M. Bell, E. P. Laine, Erosion of the Laurentide region of North America by glacial and glaciofluvial processes. *Quatern. Res.* **23**, 154–174 (1985).

3. D. E. Sugden, A case against deep erosion of shields by ice sheets. *Geology* **4**, 580–582 (1976).

4. W. A. White, Deep erosion by continental ice sheets. *Geol. Soc. Am. Bull.* **83**, 1037–1056 (1972).

5. W. E. Galloway, T. L. Whiteaker, P. Ganey-Curry, History of Cenozoic North American drainage basin evolution, sediment yield, and accumulation in the Gulf of Mexico basin. *Geosphere* **7**, 938–973 (2011).

6. A. D. Wickert, R. S. Anderson, J. X. Mitrovica, S. Naylor, E. C. Carson, The Mississippi River records glacial-isostatic deformation of North America. *Sci. Adv.* **5**, eaav2366 (2019).

7. S. S. Jamieson, D. E. Sugden, N. R. Hulton, The evolution of the subglacial landscape of Antarctica. *Earth Planet. Sci. Lett.* **293**, 1–27 (2010).

8. C. Zweck, P. Huybrechts, Modeling of the northern hemisphere ice sheets during the last glacial cycle and glaciological sensitivity. *J. Geophys. Res. Atmos.* **110**, D07103 (2005).

9. S. S. Jamieson, N. R. Hulton, M. Hagdorn, Modelling landscape evolution under ice sheets. *Geomorphology* **97**, 91–108 (2008).

10. P. U. Clark, D. Pollard, Origin of the middle Pleistocene transition by ice sheet erosion of regolith. *Paleoceanography* **13**, 1–9 (1998).

11. E. Gasson, R. M. DeConto, D. Pollard, R. H. Levy, Dynamic Antarctic ice sheet during the early to mid-Miocene. *Proc. Natl. Acad. Sci.* **113**, 3459–3464 (2016).

12. J. Austermann, D. Pollard, J. X. Mitrovica, R. Moucha, A. M. Forte, R. M. DeConto, D. B. Rowley, M. E. Raymo, The impact of dynamic topography change on Antarctic ice sheet stability during the mid-Pliocene warm period. *Geology* **43**, 927–930 (2015).

13. R. S. Anderson, M. Dühnforth, W. Colgan, L. Anderson, Far-flung moraines: Exploring the feedback of glacial erosion on the evolution of glacier length. *Geomorphology* **179**, 269–285 (2012).

14. C. L. Batchelor, M. Margold, M. Krapp, D. K. Murton, A. S. Dalton, P. L. Gibbard, C. R. Stokes, J. B. Murton, A. Manica, The configuration of Northern Hemisphere ice sheets through the Quaternary. *Nat. Commun.* **10**, 3713 (2019).

15. J. Kleman, Preservation of landforms under ice sheets and ice caps. *Geomorphology* **9**, 19–32 (1994).

16. D. E. Sugden, Glacial erosion by the Laurentide ice sheet. *J. Glaciol.* **20**, 367–391 (1978).

17. A. Melanson, T. Bell, L. Tarasov, Numerical modelling of subglacial erosion and sediment transport and its application to the North American ice sheets over the Last Glacial cycle. *Quat. Sci. Rev.* **68**, 154–174 (2013).

18. D. H. Hildes, G. K. Clarke, G. E. Flowers, S. J. Marshall, Subglacial erosion and englacial sediment transport modelled for North American ice sheets. *Quat. Sci. Rev.* **23**, 409–430 (2004).

19. M. E. Raymo, J. X. Mitrovica, M. J. O'Leary, R. M. DeConto, P. J. Hearty, Departures from eustasy in Pliocene sea-level records. *Nat. Geosci.* **4**, 328–332 (2011).

20. B. H. Wilkinson, B. J. McElroy, The impact of humans on continental erosion and sedimentation. *Geol. Soc. Am. Bull.* **119**, 140–156 (2007).

21. W. W. Shilts, Flow patterns in the central North American ice sheet. *Nature* **286**, 213–218 (1980).

22. M. S. Gauthier, T. J. Hodder, Surficial geology mapping from Manigotagan to Berens River, southeastern Manitoba (parts of NTS 62P1, 7, 8, 10, 15, 63A2, 7), in *Report of Activities* (Manitoba Geological Survey, 2020), pp. 41–46.

23. C. R. Stokes, L. Tarasov, A. S. Dyke, Dynamics of the North American Ice Sheet Complex during its inception and build-up to the Last Glacial Maximum. *Quat. Sci. Rev.* **50**, 86–104 (2012).

24. G. S. Boulton, Theory of glacial erosion, transport and deposition as a consequence of subglacial sediment deformation. *J. Glaciol.* **42**, 43–62 (1996).

25. E. Gowan, L. Niu, G. Knorr, G. Lohmann, Geology datasets in North America, Greenland and surrounding areas for use with ice sheet models. *Earth Syst. Sci. Data* **11**, 375–391 (2019).

26. F. Herman, O. Beyssac, M. Brughelli, S. N. Lane, S. Leprince, T. Adatte, J. Y. Y. Lin, J. P. Avouac, S. C. Cox, Erosion by an Alpine glacier. *Science* **350**, 193–195 (2015).

27. B. Hallet, L. Hunter, J. Bogen, Rates of erosion and sediment evacuation by glaciers: A review of field data and their implications. *Global Planet. Change* **12**, 213–235 (1996).

28. F. Herman, D. Seward, P. G. Valla, A. Carter, B. Kohn, S. D. Willett, T. A. Ehlers, Worldwide acceleration of mountain erosion under a cooling climate. *Nature* **504**, 423–426 (2013).

29. T. J. Blackburn, S. A. Bowring, J. T. Perron, K. H. Mahan, F. O. Dudas, K. R. Barnhart, An exhumation history of continents over billion-year time scales. *Science* **335**, 73–76 (2012).

30. J. K. Hillier, M. J. Smith, C. Clark, C. Stokes, M. Spagnolo, Subglacial bedforms reveal an exponential size–frequency distribution. *Geomorphology* **190**, 82–91 (2013).

31. D. Egholm, V. K. Pedersen, M. F. Knudsen, N. K. Larsen, Coupling the flow of ice, water, and sediment in a glacial landscape evolution model. *Geomorphology* **141-142**, 47–66 (2012).

32. J. Hoover Mackin, Concept of the graded river. *Geol. Soc. Am. Bull.* **59**, 463–512 (1948).

33. G. G. Roberts, N. J. White, G. L. Martin-Brandis, A. G. Crosby, An uplift history of the Colorado Plateau and its surroundings from inverse modeling of longitudinal river profiles. *Tectonics* **31**, TC4022 (2012).

34. L. Liu, The ups and downs of North America: Evaluating the role of mantle dynamic topography since the Mesozoic. *Rev. Geophys.* **53**, 1022–1049 (2015).

35. A. D. Howard, Drainage evolution in northeastern Montana and northwestern North Dakota. *Geol. Soc. Am. Bull.* **69**, 575–588 (1958).

36. J. P. Bluemle, Pleistocene drainage development in North Dakota. *Geol. Soc. Am. Bull.* **83**, 2189–2193 (1972).

37. M. A. Kessler, R. S. Anderson, J. P. Briner, Fjord insertion into continental margins driven by topographic steering of ice. *Nat. Geosci.* **1**, 365–369 (2008).

38. S. Bukhari, N. Eyles, S. Sookhan, R. Mulligan, R. Paulen, M. Krabbendam, N. Putkinen, Regional subglacial quarrying and abrasion below hard-bedded palaeo-ice streams crossing the Shield–Palaeozoic boundary of central Canada: The importance of substrate control. *Boreas* **50**, 781–805 (2021).

39. J. W. Spencer, Origin of the basins of the Great Lakes of America. *Quart. J. Geol. Soc.* **46**, 523–533 (1890).

40. M. Ross, J. E. Campbell, M. Parent, R. S. Adams, Palaeo-ice streams and the subglacial landscape mosaic of the North American mid-continental prairies. *Boreas* **38**, 421–439 (2009).

41. P. M. Cutler, D. M. Mickelson, P. M. Colgan, D. R. MacAyeal, B. R. Parizek, Influence of the Great Lakes on the dynamics of the southern Laurentide Ice Sheet: Numerical experiments. *Geology* **29**, 1039–1042 (2001).

42. L. E. Lisiecki, M. E. Raymo, A Pliocene-Pleistocene stack of 57 globally distributed benthic $\delta^{18}O$ records. *Paleoceanography* **20**, PA1003 (2005).

43. L. S. Anderson, G. H. Roe, R. S. Anderson, The effects of interannual climate variability on the moraine record. *Geology* **42**, 55–58 (2014).

44. B. A. Lusardi, C. E. Jennings, K. L. Harris, Provenance of Des Moines lobe till records ice-stream catchment evolution during Laurentide deglaciation. *Boreas* **40**, 585–597 (2011).

45. E. J. Gowan, X. Zhang, S. Khosravi, A. Rovere, P. Stocchi, A. L. C. Hughes, R. Gyllencreutz, J. Mangerud, J. I. Svendsen, G. Lohmann, A new global ice sheet reconstruction for the past 80 000 years. *Nat. Commun.* **12**, 1199 (2021).

46. M. Margold, C. R. Stokes, C. D. Clark, Ice streams in the Laurentide Ice Sheet: Identification, characteristics and comparison to modern ice sheets. *Earth Sci. Rev.* **143**, 117–146 (2015).

47. B. Hallet, Glacial quarrying: A simple theoretical model. *Ann. Glaciol.* **22**, 1–8 (1996).

48. C. Gao, J. Shirota, R. I. Kelly, F. R. Brunton, S. Van Haaften, Bedrock topography and overburden thickness mapping, southern Ontario (Misc. Data Release 207, Ontario Geological Survey, 2006); www.geologyontario.mndm.gov.on.ca/mndmaccess/mndm_dir.asp?type=pub&id=MRD207.

49. M. F. Hutchinson, A new procedure for gridding elevation and stream line data with automatic removal of spurious pits. *J. Hydrol.* **106**, 211–232 (1989).

50. M. F. Hutchinson, T. I. Dowling, A continental hydrological assessment of a new grid-based digital elevation model of Australia. *Hydrol. Process.* **5**, 45–58 (1991).

51. M. F. Hutchinson, T. Xu, J. A. Stein, Recent progress in the ANUDEM elevation gridding procedure. *Geomorphometry* **2011**, 19–22 (2011).

52. J. Kirby, C. Swain, A reassessment of spectral T_e estimation in continental interiors: The case of North America. *J. Geophys. Res. Solid Earth* **114**, B08401 (2009).

53. M. Tesauro, M. K. Kaban, S. A. Cloetingh, Global strength and elastic thickness of the lithosphere. *Global Planet. Change* **90-91**, 51–57 (2012).

54. W. R. Peltier, Global glacial isostasy and the surface of the ice-age Earth: The ICE-5G (VM2) model and GRACE. *Annu. Rev. Earth Planet. Sci.* **32**, 111–149 (2004).

55. D. F. Argus, W. Peltier, R. Drummond, A. W. Moore, The Antarctica component of postglacial rebound model ICE-6G_C (VM5a) based on GPS positioning, exposure age dating of ice thicknesses, and relative sea level histories. *Geophys. J. Int.* **198**, 537–563 (2014).

56. W. R. Peltier, R. Drummond, Rheological stratification of the lithosphere: A direct inference based upon the geodetically observed pattern of the glacial isostatic adjustment of the North American continent. *Geophys. Res. Lett.* **35**, L16314 (2008).

57. B. Hager, M. Richards, Long-wavelength variations in Earth's geoid: Physical models and dynamical implications. *Philos. Trans. R. Soc. Lond. A* **328**, 309–327 (1989).

58. H. Hobbs, Origin of the Driftless Area by subglacial drainage-A new hypothesis. *Geol. Soc. Am. Spec. Paper* **337**, 93–102 (1999).

59. G. N. Coffey, Major glacial drainage changes in Ohio. *Ohio J. Sci.* **58**, 43–49 (1958).

60. R. L. Rieck, H. A. Winters, Characteristics of a glacially buried cuesta in southeast Michigan. *Annals Assoc. Am. Geograph.* **72**, 482–494 (1982).

61. A. J. Shunk, S. G. Driese, J. O. Farlow, M. S. Zavada, M. K. Zobaa, Late Neogene paleoclimate and paleoenvironment reconstructions from the Pipe Creek Sinkhole, Indiana, USA. *Palaeogeogr. Palaeoclimatol. Palaeoecol.* **274**, 173–184 (2009).

62. M. E. McMillan, C. L. Angevine, P. L. Heller, Postdepositional tilt of the Miocene-Pliocene Ogallala Group on the western Great Plains: Evidence of late Cenozoic uplift of the Rocky Mountains. *Geology* **30**, 63–66 (2002).

63. M. Margold, C. R. Stokes, C. D. Clark, J. Kleman, Ice streams in the Laurentide Ice Sheet: A new mapping inventory. *J. Maps* **11**, 380–395 (2015).

64. A. S. Dalton, M. Margold, C. R. Stokes, L. Tarasov, A. S. Dyke, R. S. Adams, S. Allard, H. E. Arends, N. Atkinson, J. W. Attig, P. J. Barnett, R. L. Barnett, M. Batterson, P. Bernatchez, H. W. Borns Jr, A. Breckenridge, J. P. Briner, E. Brouard, J. E. Campbell, A. E. Carlson, J. J. Clague, B. B. Curry, R. A. Daigneault, H. Dubé-Loubert, D. J. Easterbrook, D. A. Franzi, H. G. Friedrich, S. Funder, M. S. Gauthier, A. S. Gowan, K. L. Harris, B. Hétu, T. S. Hooyer, C. E. Jennings, M. D. Johnson, A. E. Kehew, S. E. Kelley, D. Kerr, E. L. King, K. K. Kjeldsen, A. R. Knaeble, P. Lajeunesse, T. R. Lakeman, M. Lamothe, P. Larson, M. Lavoie, H. M. Loope, T. V. Lowell, B. A. Lusardi, L. Manz, I. McMartin, F. C. Nixon, S. Occhietti, M. A. Parkhill, D. J. W. Piper, A. G. Pronk, P. J. H. Richard, J. C. Ridge, M.

Ross, M. Roy, A. Seaman, J. Shaw, R. R. Stea, J. T. Teller, W. B. Thompson, L. H. Thorleifson, D. J. Utting, J. J. Veillette, B. C. Ward, T. K. Weddle, H. E. Wright Jr, An updated radiocarbon-based ice margin chronology for the last deglaciation of the North American Ice Sheet Complex. *Quat. Sci. Rev.* **234**, 106223 (2020).

65. H. H. Gray, Origin and history of the Teays drainage system: The view from midstream. *Geol. Soc. Am. Spec. Paper* **258**, 43–50 (1991).

66. M. J. Guccione, K. Mueller, J. Champion, S. Shepherd, S. D. Carlson, B. Odhiambo, A. Tate, Stream response to repeated coseismic folding, Tiptonville dome, New Madrid seismic zone. *Geomorphology* **43**, 313–349 (2002).

67. S. Naylor, J. Schumacher, B. J. Sperl, Map showing elevation of the bedrock surface in Indiana (Misc. Map 94A, IN Geological Survey, 2016); http://maps.indiana.edu/previewMaps/Geology/Bedrock_Surface_Contours.html.

68. M. A. Jirsa, T. J. Boerboom, V. W. Chandler, J. H. Mossler, A. C. Runkel, D. R. Setterholm, Minnesota Bedrock Topography (Online Map Services, Univ. of MN, 2011); https://mngs-umn.opendata.arcgis.com/maps/UMN::bedrock-topography-2016/explore?location=45.703396%2C-93.822900%2C6.00.

69. J. A. Wilhelm, "A GIS comparison of surficial and bedrock-simulated stream flow in the glaciated Appalachian Plateau, Northwestern Pennsylvania," thesis, Allegheny College, Meadville, PA (2017).

70. National Geophysical Data Center, *Bathymetry of Lake Michigan* (National Geophysical Data Center, NOAA, 1996).

71. A. Gronewold, V. Fortin, B. Lofgren, A. Clites, C. A. Stow, F. Quinn, Coasts, water levels, and climate change: A Great Lakes perspective. *Clim. Change* **120**, 697–711 (2013).

72. B. L. Herzog, B. J. Stiff, C. A. Chenoweth, K. L. Warner, J. B. Sieverling, C. Avery, *Buried Bedrock Surface of Illinois* (IL Map 5, IL Geological Survey, 1994); http://clearinghouse.isgs.illinois.edu/data/geology/bedrock-topography.

73. C. S. McGarry, *Bedrock Topography of Caroll County, Illinois* (OF Map 1997-13e, IL Geological Survey, 1997).

74. C. S. McGarry, M. H. Riggs, *Bedrock Surface Topography Map, Jo Daviess County, Illinois* (OF Map 2000-8e, IL State Geological Survey, 2000).

75. OH Division of Geological Survey, *Bedrock-Topography Data for Ohio* (Digital Map Series BG-3, OH Division of Geological Survey, 2003).

76. Soil Survey Staff, *The Gridded Soil Survey Geographic* (*gSSURGO*) *Database for Ohio* (USDA Natural Resources Conservation Service, 2016).

77. WI Dept. of Natural Resources, Groundwater contamination susceptibility in Wisconsin (WI GW Mgmt. Plan: Report No. 5, WI Dept. of Natural Resources, 1986); https://data-wi-dnr.opendata.arcgis.com/datasets/gcsm-bedrock-depth.

78. Ontario Geological Survey, Ontario's Quaternary geology at a compilation scale of 1:1,000,000 (Ontario Ministry of Northern Development, Natural Resources and Forstry, 2000); www.geologyontario.mndm.gov.on.ca/mndmaccess/mndm_dir.asp?type=pub&id=eds014-rev.

79. B. H. Feenstra, *Bedrock topography of the Niagara and Niagara-on-the-Lake Area* (Map P.2400, Ontario Geological Survey, 1981).

80. B. H. Feenstra, *Bedrock topography of the Grimsby Area, Southern Ontario* (Map P.2401, Ontario Geological Survey 1981).

81. D. Hutchinson, C. F. Lewis, G. Hund, Regional stratigraphic framework of surficial sediments and bedrock beneath Lake Ontario. *Geogr. Phys. Quatern.* **47**, 337–352 (1993).

82. National Geophysical Data Center, *Bathymetry of Lake Huron* (National Geophysical Data Center, NOAA, 1999).

83. C. Gao, Buried bedrock valleys and glacial and subglacial meltwater erosion in southern Ontario, Canada. *Can. J. Earth Sci.* **48**, 801–818 (2011).

84. J. R. Hoaglund, J. J. Kolak, D. T. Long, G. J. Larson, Analysis of modern and Pleistocene hydrologic exchange between Saginaw Bay (Lake Huron) and the Saginaw Lowlands area. *Geol. Soc. Am. Bull.* **116**, 3–15 (2004).

85. National Geophysical Data Center, *Bathymetry of Lake Erie and Lake Saint Clair* (National Geophysical Data Center, NOAA, 1999).

86. R. J. Wold, D. R. Hutchinson, T. C. Johnson, 14: Topography and surficial structure of Lake Superior bedrock as based on seismic reflection profiles. *Geol. Soc. Am. Mem.* **156**, 257–272 (1982).

87. NOAA National Centers for Environmental Information, Bathymetry of Lake Superior (2021); www.ngdc.noaa.gov/mgg/greatlakes/superior.html.

88. R. J. Schaetzl, H. Enander, M. D. Luehmann, D. P. Lusch, C. Fish, M. Bigsby, M. Steigmeyer, J. Guasco, C. Forgacs, A. Pollyea, Mapping the physiography of Michigan with GIS. *Phys. Geography* **34**, 2–39 (2013).

89. KY Geological Survey, The KY Groundwater Data Repository (University of Kentucky, 2021); http://kgs.uky.edu/kgsweb/DataSearching/watersearch.asp.

90. B. J. Witzke, R. R. Anderson, J. P. Pope, Iowa Bedrock Topography (Open File Map OFM-2010-1, Iowa Geological and Water Survey, 2010); https://ir.uiowa.edu/igs_ofm/71/.

91. N. Atkinson, S. Lyster, Bedrock Topography of Alberta, Canada (Map 550, Alberta Geological Survey, 2010); http://ags.aer.ca/publications/MAP_550.html.

92. D. W. Tomhave, L. D. Schulz, Bedrock geologic map showing configuration of the bedrock surface in South Dakota east of the Missouri River (General Map G-09, South Dakota Geological Survey, 2004); http://www.sdgs.usd.edu/pubs/pdf/ESDBedrock_20040630.zip.

93. Saskatchewan Water Security Agency, Mapping: Mapsheets (Saskatchewan Water Security Agency, 2021); www.wsask.ca/Water-Info/Ground-Water/Mapping/.

94. R. R. Burchett, V. H. Dreeszen, E. C. Reed, *Bedrock Geologic Map Showing Thickness of Overlying Quaternary Deposits, Lincoln Quadrangle and Part of Nebraska City Quadrangle, Nebraska and Kansas* (USGS Map I-729, 1972).

95. R. R. Burchett, E. C. Reed, V. H. Dreeszen, *Bedrock Geologic Map Showing Thickness of Overlying Quaternary Deposits, Freemont Quadrangle and part of Omaha Quadrangle, Nebraska* (USGS Map I-905, 1975).

96. R. R. Burchett, V. H. Dreeszen, V. L. Souders, G. E. Prichard, Bedrock geologic map showing configuration of the bedrock surface in the Nebraska part of the Sioux City Quadrangle (USGS Map I-1879, 1988).

97. R. N. Bergantino, Topographic map of the bedrock surface, Northeastern Montana, Northwestern North Dakota, and Southeastern Saskatchewan (OF Map 151, MT Bureau of Mines and Geology, 1984).

98. J. P. Bluemle, Geologic and topographic bedrock map of North Dakota (Misc. Map 25, ND Geological Survey, 1983).

99. J. E. Denne, R. E. Miller, L. R. Hathaway, H. G. O'Conner, W. C. Johnson, Hydrogeology and geochemistry of glacial deposits in Northeastern Kansas (Bulletin 229, KS Geological Survey, 1998).

100. G. E. Heim, W. B. Howe, Map of the bedrock topography of Northwestern Missouri (Groundwater Maps of MO, MO Div. of Geological Survey and Water Resources, 1962).

101. MO Dept. of Natural Resources, Geologic Well Logs of Missouri (MO Dept. of Natural Resources, 2021); https://dnr.mo.gov/geology/wrc/logmain/index.html?/env/wrc/logmain/index.html.

102. Manitoba Geological Survey, 3-D Geological Mapping in Manitoba: Moving Forward (Manitoba Geological Survey, 2021); www.manitoba.ca/iem/geo/3dmodel/index.html.

103. B. B. Bannatyne, R. W. Klassen, J. E. Wyder, Bedrock topography and geology of southern Manitoba (PM 25, Geological Survey of Canada, 1970).

104. G. L. D. Matile, G. R. Keller, Surficial geology of southern Manitoba (south of 53°) (Surficial Geology Compilation Map Series No. SG-SMB, Manitoba Innovation, Energy and Mines, Manitoba Geological Survey, 2004).

105. W. Tobler, Resolution, resampling, and all that, in *Building Databases for Global Science* (Taylor and Francis, London, 1988), pp. 129–137.

106. T. G. Farr, M. Kobrick, Shuttle Radar Topography Mission produces a wealth of data. *Eos* **81**, 583–585 (2000).

107. B. J. Zaprowski, E. B. Evenson, F. J. Pazzaglia, J. B. Epstein, Knickzone propagation in the Black Hills and northern High Plains: A different perspective on the late Cenozoic exhumation of the Laramide Rocky Mountains. *Geology* **29**, 547–550 (2001).

108. R. P. Goldthwait, The Teays Valley problem; A historical perspective, in *Geology and Hydrogeology of the Teays-Mahomet Bedrock Valley System: Boulder, CO*, W. N. Melhorn, J. P. Kempton, Eds. (Geological Society of America Special Papers 258, 1991), pp. 3–8.

109. D. A. Leckie, Tertiary fluvial gravels and evolution of the Western Canadian Prairie landscape. *Sediment. Geol.* **190**, 139–158 (2006).

110. D. I. Cummings, H. A. Russell, D. R. Sharpe, Buried-valley aquifers in the Canadian Prairies: Geology, hydrogeology, and origin. *Can. J. Earth Sci.* **49**, 987–1004 (2012).

111. M. Fenton, B. Schreiner, E. Nielsen, J. Pawlowicz, Quaternary geology of the Western Plains (Geological Atlas of the Western Canada Sedimentary Basin, Canadian Society of Petroleum Geologists and Alberta Research Council, 1994).

112. A. Duk-Rodkin, O. L. Hughes, Tertiary-Quaternary drainage of the pre-glacial Mackenzie Basin. *Quat. Int.* **22-23**, 221–241 (1994).

113. J. W. Sears, Late Oligocene–Early Miocene Grand Canyon: A Canadian connection. *GSA Today* **23**, 4–10 (2013).

114. L. Horberg, R. C. Anderson, Bedrock topography and Pleistocene glacial lobes in central United States. *J. Geol.* **64**, 101–116 (1956).

115. G. Larson, R. Schaetzl, Origin and evolution of the Great Lakes. *J. Great Lakes Res.* **27**, 518–546 (2001).

116. T. C. Chamberlin, F. Leverett, ART. XXIV.—Further studies of the drainage features of the Upper Ohio Basin. *Am. J. Sci.* **47**, 247 (1894).

117. W. G. Tight, *Drainage Modifications in Southeastern Ohio and Adjacent Parts of West Virginia and Kentucky* (U.S. Government Printing Office, 1903).

118. J. T. Teller, R. P. Goldthwait, The old Kentucky River; a major tributary to the Teays River. *Geol. Soc. Am. Spec. Paper* **258**, 29–42 (1991).

119. S. J. Luft, The South Fork of the Licking River; eastern Kentucky's major late Tertiary river. *Southeastern Geol.* **26**, 239–249 (1986).

120. W. J. Wayne, Pleistocene evolution of the Ohio and Wabash valleys. *J. Geol.* **60**, 575–585 (1952).

121. N. Eyles, E. Arnaud, A. E. Scheidegger, C. H. Eyles, Bedrock jointing and geomorphology in southwestern Ontario, Canada: An example of tectonic predesign. *Geomorphology* **19**, 17–34 (1997).

122. W. M. Merrill, Pleistocene history of a part of the Hocking River Valley, Ohio (Report of Investigations No. 16, OH Div. of Geological Survey, 1953).

123. R. B. Jacobson, D. P. Elston, J. W. Heaton, Stratigraphy and magnetic polarity of the high terrace remnants in the upper Ohio and Monongahela rivers in West Virginia, Pennsylvania, and Ohio. *Quatern. Res.* **29**, 216–232 (1988).

124. A. M. Sarna-Wojcicki, M. S. Pringle, J. Wijbrans, New 40Ar/39Ar age of the Bishop Tuff from multiple sites and sediment rate calibration for the Matuyama-Brunhes boundary. *J. Geophys. Res. Solid Earth* **105**, 21431–21443 (2000).

125. D. E. Granger, D. Fabel, A. N. Palmer, Pliocene−Pleistocene incision of the Green River, Kentucky, determined from radioactive decay of cosmogenic ^{26}Al and ^{10}Be in Mammoth Cave sediments. *Geol. Soc. Am. Bull.* **113**, 825–836 (2001).

126. D. J. Ward, J. A. Spotila, G. S. Hancock, J. M. Galbraith, New constraints on the late Cenozoic incision history of the New River, Virginia. *Geomorphology* **72**, 54–72 (2005).

127. G. A. Galgana, M. W. Hamburger, Geodetic observations of active intraplate crustal deformation in the Wabash Valley seismic zone and the southern Illinois basin. *Seismol. Res. Lett.* **81**, 699–714 (2010).

128. J. E. Todd, The Pleistocene history of the Missouri River. *Science* **39**, 263–274 (1914).

129. E. D. McKay, Quaternary deposits and history of the ancient Mississippi River valley, north-central Illinois. Open file series 2005-07 (2005).

130. J. C. Knox, J. W. Attig, Geology of the pre-Illinoian sediment in the Bridgeport Terrace, lower Wisconsin River Valley, Wisconsin. *J. Geol.* **96**, 505–514 (1988).
